# Benchmarking Membrane Protein Detergent Stability for Improving Throughput of High-Resolution X-ray Structures

**DOI:** 10.1016/j.str.2010.12.001

**Published:** 2011-01-12

**Authors:** Yo Sonoda, Simon Newstead, Nien-Jen Hu, Yilmaz Alguel, Emmanuel Nji, Konstantinos Beis, Shoko Yashiro, Chiara Lee, James Leung, Alexander D. Cameron, Bernadette Byrne, So Iwata, David Drew

**Affiliations:** 1 Division of Molecular Biosciences, Membrane Protein Crystallography Group, Imperial College, London SW7 2AZ, UK; 2 Membrane Protein Laboratory, Diamond Light Source, Harwell Science and Innovation Campus, Didcot, Chilton, Oxfordshire OX11 ODE, UK; 3 Japan Science and Technology Agency, ERATO, Human Receptor Crystallography Project, Yoshida Konoe, Sakyo-ku, Kyoto 606-8501, Japan

## Abstract

Obtaining well-ordered crystals is a major hurdle to X-ray structure determination of membrane proteins. To facilitate crystal optimization, we investigated the detergent stability of 24 eukaryotic and prokaryotic membrane proteins, predominantly transporters, using a fluorescent-based unfolding assay. We have benchmarked the stability required for crystallization in small micelle detergents, as they are statistically more likely to lead to high-resolution structures. Using this information, we have been able to obtain well-diffracting crystals for a number of sodium and proton-dependent transporters. By including in the analysis seven membrane proteins for which structures are already known, AmtB, GlpG, Mhp1, GlpT, EmrD, NhaA, and LacY, it was further possible to demonstrate an overall trend between protein stability and structural resolution. We suggest that by monitoring membrane protein stability with reference to the benchmarks described here, greater efforts can be placed on constructs and conditions more likely to yield high-resolution structures.

## Graphical abstract

**Graphical Abstract:**

**Figure:**
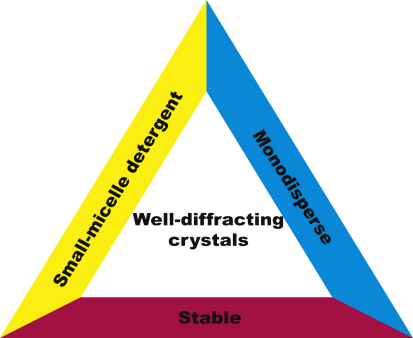

**Highlights:**

► Benchmarked the stability required for crystallization in small sized detergents ► Membrane protein stability is inherent to the protein rather than detergent specific ► Membrane proteins stable in LDAO are more likely to yield well-diffracting crystals ► Eukaryotic membrane proteins are 3-fold less stable in 12M than prokaryotic proteins

## Introduction

Structural determination of integral membrane proteins represents a major challenge. Integral membrane proteins make up more than a quarter of all sequenced genomes (Wallin and von Heijne, 1998) and yet, to date, only around 180 unique polytopic structures have been elucidated (http://blanco.biomol.uci.edu/). Transport proteins make up a large fraction of the membrane proteome, and although numerous studies have shown that transporters play key roles in drug pharamacokinetics (Giacomini et al., 2010), only two mammalian structures have been solved (Aller et al., 2009; Pebay-Peyroula et al., 2003). These low transporter numbers are a reflection of the technical difficulties of working with membrane proteins. As one might expect, tools that can reduce the time it takes to solve novel membrane protein crystal structures are in great demand.

C-terminal tagging of membrane proteins with green fluorescent protein (GFP) facilitates monitoring of both expression and purification of target proteins (Drew et al., 2001, 2005). GFP fluorescence from whole-cell culture and from SDS-polyacrylamide gels provides a direct measure of the amount of membrane-integrated expression (Drew et al., 2006) while “monodispersity” screening, by fluorescence-detection size-exclusion chromatography (FSEC), makes it possible to evaluate the quality of the expressed fusion using crude detergent solubilized extracts prior to purification (Kawate and Gouaux, 2006). Selected homologs can be grown for large-scale culturing and purification by Ni^2+^-affinity chromatography by including a His_6-10_-tag on GFP. Subsequent cleavage with a site-specific protease to remove the GFP-His_6-10_ fusion, followed by a second step of Ni^2+^-affinity chromatography, allows isolation of the untagged protein (Drew et al., 2001, 2005). For these reasons, there is an increasing number of membrane protein structures solved using material expressed and purified with a protease cleavable GFP-His_6-10_ fusion tag: zebrafish ATP-gated P2X(4) receptor (Kawate et al., 2009), chicken acid-sensing ion-channel (ASIC) (Jasti et al., 2007), bacterial proton-coupled broad-specificity amino acid transporter ApcT (Shaffer et al., 2009), rat ionotropic AMPA-glutamate receptor GluA2 (Sobolevsky et al., 2009), chicken strong inward-rectifier potassium channel Kir2.2 (Tao et al., 2009), bacterial carnitine transporter CaiT (Tang et al., 2010), and red algae ClC transporter CmCLC (Feng et al., 2010).

Although pipelines such as the one described here can greatly facilitate overexpression and purification screening, arguably the biggest obstacle to structure determination is crystal optimization. It is generally thought that the membrane proteins that remain stable in different detergent solutions are more likely to form better ordered crystals. However, the effect of membrane protein stability on crystal growth has not been systematically studied.

To provide some quantitative measures we have determined the stability of 17 pro- and eukaryotic transport proteins using a fluorescent-based unfolding assay. We have analyzed this data in relation to the quality of crystals obtained, defined by how well crystals could be optimized to diffract X-rays to high-resolution. As additional controls, seven membrane proteins whose structures are already known were also included in this analysis. Our findings show that that there is indeed a measurable correlation between the stability of the membrane protein in detergent solution and the likelihood of obtaining well-ordered crystals. Using this information, we have been able to obtain X-ray structures for a number of secondary active transporters.

## Results and Discussion

### GFP-Based Pipeline Strategy for Target Selection, Purification, and Crystallization of Eukaryotic and Prokaryotic Transporters

Previous screening of eukaryotic membrane proteins in *Saccharomyces cerevisiae* shows that only a quarter can be overexpressed to levels suitable for structural studies (Newstead et al., 2007). For this reason, we started by cloning a large number of eukaryotic transporters ∼140, taken from 12 different transporter families using cDNA from *Oryza sativia*, *Arabadopsis thaliana*, *Mus musculus*, *Rattus novergicus*, *or Homo sapiens*, into the GFP-His_8_ containing 2μ vector pDDGFP-2. The cloned targets were screened in *S. cerevisiae* for full-length expression greater than 1 mg.l^-1^ using whole-cell and in-gel fluorescence, as described previously (Newstead et al., 2007). Using the same criterion, we cloned and screened 40 bacterial transporters in *Escherichai coli*, of which most are homologs of those screened in *S. cerevisiae*, plus five archeal ABC transporters and six antibiotic resistance proteins that share no eukaryotic sequence homology (Drew et al., 2006). Bacterial and eukaryotic transporters that displayed a sharp symmetrical FSEC elution profile in dodecyl-β-D-maltopyranoside (12M) were subsequently grown in large-scale cultures for purification and crystallization trials. As summarized in Table 1A, we selected 47 transporters based on overexpression, in-gel fluorescence and FSEC profiles for submission to purification and crystallization trials.

**Figure 1:**
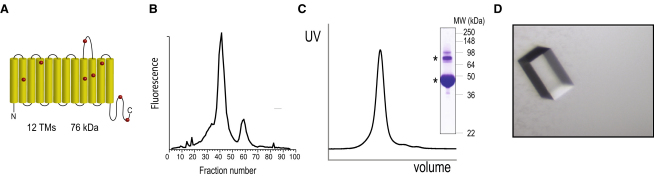
Example of the Experimental Data Measured for Each Membrane Protein Crystallized in the Detergent 12M (A) Topology prediction of the rice anion exchanger (PT-2) with the position of the cysteines depicted as red spheres. (B) FSEC trace of PT-2 in 12M-solubilized membranes. (C) SEC trace of purified PT-2 and SDS-PAGE analysis of the pooled peak shown in the upper right panel; asterisk for PT-2 protein which migrates as two separate bands in the gel as confirmed by mass-spectrometry. (D) Crystal of PT-2 in the detergent 12M obtained after optimization (see Figures S1A and S1B for transporters BT1-9, MT1-4, and PT1-4; and Figure S2).

In total, 17 of the 47 pro- and eukaryotic transporters purified could be crystallized: 8 bacterial transporters (BT-1 to BT-8), 1 archeal (AT-1) and 8 eukaryotic transport proteins (PT-1 to PT-4 and MT-1 to MT-4) (Figure 1; see Figures S1A and S1B available online). An average crystallization success rate of ∼35% for both bacterial and eukaryotic transporters is comparable to that obtained for globular proteins among structural genomics consortia (Price et al., 2009).

The majority of transporter crystals diffracted X-rays between 8 and 10 Å, Table 1B. After standard crystallographic techniques to try and improve crystal quality, only the eukaryotic transporter PT-2 and the bacterial transporters BT-2 and BT-3 could be optimized to diffract X-rays to 4 Å resolution or higher (Table 1B; Figure S2A). The best X-ray diffraction obtained by the other transporter crystals was no greater than 5 Å. None of the mammalian transporter crystals diffracted X-rays higher than 8 Å resolution (MT1 to MT4).

### Crystal Optimization of Control Membrane Proteins

Although 12M has been the most successful crystallization detergent for α-helical membrane proteins (Newstead et al., 2008), it forms large micelles which can hinder crystal contacts (Michel, 2001). To confirm that 12M is responsible for the poor quality of the target transporter crystals, we repeated the crystallization for seven other membrane proteins whose structures are already known: LacY, lactose permease (Abramson et al., 2003); AmtB, ammonium channel (Zheng et al., 2004); GlpG, rhomboid protease (Wang et al., 2006); NhaA, sodium-proton exchanger (Hunte et al., 2005); GlpT, glycerol-3-phosphate transporter (Huang et al., 2003); EmrD, multidrug transporter (Yin et al., 2006), and Mhp1, hydantoin transporter (Weyand et al., 2008). To avoid biasing the analysis, we followed our standard purification procedure outlined in Experimental Procedures for each of these control proteins.

All our control proteins formed crystals in 12M using the sparse-matrix screen MemGold (Figure S1C). As observed for our target transporters, initial diffraction was also poor with typical diffraction limits of ∼4–8 Å resolution (Table 1B). In contrast, by repeating the purification of AmtB in n-dodecyl-N,N-dimthylamine-N-oxide (LDAO), we obtained crystals with a comparable quality to the reported structure, diffracting to 1.9 Å (Figure S2B). Similarly, Mhp1 crystals grown in n-nonyl-β-D-maltopyranoside (9M) diffracted X-rays equivalent to those published at 2.8 Å (Figure S2B). Although it proved impossible to grow crystals of GlpG in nonyl-β-D-glucopyranoside (NG), the protein we used for analysis is the full-length protein, whereas the published structures are of the core protein lacking 87 amino acids from the N terminus (Wang et al., 2006). Nonetheless, after further trials, full-length GlpG crystals were grown in n-decyl-β-D-maltopyranoside (10M) that diffracted anisotropically to ∼3.5 Å (Figure S2B). These results strongly suggest that using the detergent 12M is the main reason for poor crystal quality. However, to confirm that protein produced in our pipeline does not adversely affect crystal quality, crystals for one of the control proteins (NhaA) were also optimized in 12M.

After ten purifications from 5 liter cell-culture preparations, NhaA crystals could be obtained that diffract X-rays to 3.5 Å resolution in the best direction (Figure S2B). The arrangement of NhaA in these crystals represents the physiological dimer, in contrast to the nonphysiological monomeric X-ray structure (Hunte et al., 2005; unpublished data). This side-by-side arrangement of NhaA is consistent with that obtained by EM and ESR (Appel et al., 2009; Hilger et al., 2007). In addition to NhaA, we have also compared the crystallization of a bacterial transporter (oligopeptide transporter, PepT_So_) as a C-terminal His_6_-tagged construct and as an untagged construct from the GFP pipeline (Newstead et al., 2010). While the PepT_So_-His_6_ crystals do not diffract X-rays beyond 4–5 Å, even after extensive optimization, crystals grown using protein from our GFP-based pipeline diffract to 3.6 Å resolution in the best direction (Figures S1A and S2C). It is plausible that this difference is due to the presence of the C-terminal His_6_ tag, which may hinder the formation of well-ordered PepT_So_ crystals. The diffraction data obtained from the untagged construct has enabled us to solve the structure of PepT_So_ (Newstead et al., 2010); added to our data set as BT-9 (Table 1B).

### Benchmarking Membrane Protein Stability in Detergent Solutions

In agreement with this analysis, statistically it has been shown that α-helical membrane proteins that crystallize in 12M produce, on average, lower resolution structures (Sonoda et al., 2010). In particular, secondary-active transporters that lack large hydrophilic domains tend to yield 3–4 Å resolution structures in 12M, which is insufficient if we are to fully understand mechanistic details (Sonoda et al., 2010). Small micelle detergents are an alternative to 12M; however, they represent a much harsher environment for membrane proteins. Studies have suggested that the chance of obtaining crystals in small detergents is higher if the protein is more thermostable (Serrano-Vega et al., 2008). The best documented example is stabilization of the turkey β_1_-adrenergic receptor by alanine scanning mutagenesis (Serrano-Vega et al., 2008). The thermostabilized receptor, with a melting temperature 21°C higher than the native sequence, crystallized in the short-chain detergent octylthioglucoside. The GPCR structure in this detergent was solved to 2.7 Å resolution (Warne et al., 2008).

**Figure 2:**
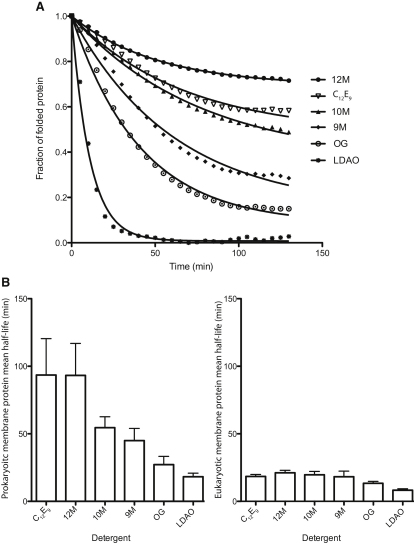
Membrane Protein Stability as Quantified by the CPM Assay (A) Example of the unfolding curves measured for each membrane protein at 40°C for 130 min shown here for the bacterial transporter BT-4 in the following detergents: 12M, filled circle; 10M, filled triangle; 9M, filled diamond; LDAO, asterisk; C_12_E_9_, open triangle; OG, open circle. (B) Mean unfolding rates for n = 16 bacterial (left panel) and n = 8 eukaryotic membrane proteins (right panel) in all detergents C_12_E_9,_ 12M, 10M, 9M, LDAO except OG where n = 12 and 6, respectively. The unfolding half-life for each protein was calculated by fitting the data from the CPM assay to a single exponential decay function as described previously (Roth et al., 2008). See also Figures S3 and S4.

One critical question is how stable does a given membrane protein have to be in a small sized detergent to crystallize? To address this conundrum, we measured the stability of the target transporters and control proteins in six of the most successful crystallization detergents using a fluorescent-based unfolding assay (Alexandrov et al., 2008). In this assay the dye N-[4-(7-diethylamino-4-methyl-3-coumarinyl)phenyl]-maleimide (CPM) principally becomes fluorescent upon reacting with free sulfhydryl groups. As most cysteines are predominantly located within TM segments, cysteine accessibility is a good measure of protein unfolding (Alexandrov et al., 2008). All transporters in our sample set, apart from AT-1, BT-2 and BT-8, were predicted to have one or more transmembrane cysteine(s) (Krogh et al., 2001) (Figures S1A–S1C). The ABC transporter AT-1 was therefore omitted from this analysis, while for BT-2 and BT-8 single cysteine point mutants were measured instead (these cysteine mutants yield crystals that diffract X-rays comparable to wild-type protein). Protein at ∼10 mg.ml^-1^ in 0.03% 12M was diluted 150-fold into a Tris-HCl buffer (pH 7.5) containing each of the following detergents: 12M, 10M, 9M, LDAO, dodecyl nonaethylene glycol ether (C_12_E_9_) or n-octyl-β-D-glucopyranside (OG) (see Experimental Procedures for further details). After incubation in the detergent buffer for 5 min, we monitored unfolding using a 96-well spectrofluoremeter for 130 min at a single temperature of 40°C (Figure S3). By calculating the fraction of folded protein at each time point, we fitted a single exponential decay curve (Figure 2A) as outlined previously (Roth et al., 2008). Typically the highest CPM fluorescence over this time period, taken as the “maximal” unfolded state, was observed in the detergent LDAO.

As shown in Figure 2B, the mean stability of the bacterial transporters, with a half-life (t_1/2_) = 93 min, is longest in the detergents 12M (72 kDa) and C_12_E_9_ (83 kDa) with the largest micelle sizes (Strop and Brunger, 2005). The t_1/2_ is shorter as the micelle-size of the detergents decreases, 10M (39 kDa) t_1/2_ = 54 min, 9M (31 kDa) t_1/2_ = 45 min, OG (26 kDa) t_1/2_ = 27 min and LDAO (17 kDa) t_1/2_ = 18 min (le Maire et al., 2000; Bamber et al., 2007; Strop and Brunger, 2005). Indeed, the mean unfolding rates of the bacterial transporters correlate linearly with the micelle size of the detergents, R^2^ = 0.95 (Figure S4). Strikingly, the mean stability of the eukaryotic transporters is several-fold lower than their bacterial counterparts in these detergents, 12M t_1/2_ = 21 min, C_12_E_9_ t_1/2_ = 18 min, 10M t_1/2_ = 19 min, 9M t_1/2_ = 18, OG t_1/2_ = 12 min and LDAO t_1/2_ = 8 (Figure 2B). However, because BT-1, BT-8, BT-3, MT-1, and PT-4 transporters visually precipitated in OG we did not include their unfolding values in this analysis. This may have resulted in some bias, and because this observation adds some uncertainly as to the validity of the other measurements in OG we decided not to use the data from this detergent in any further analysis. To verify the reliability of the t_1/2_ estimates measured in the other detergents, we compared the unfolding rates in LDAO to the FSEC profiles in this detergent. LDAO was selected as it is the harshest of the six detergents screened.

**Figure 3:**
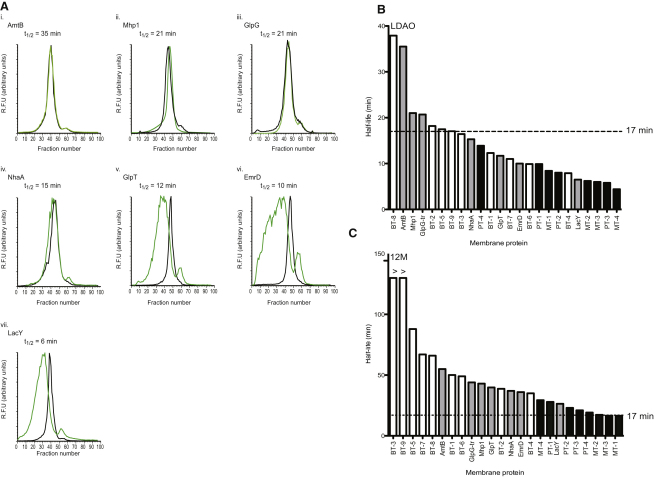
Benchmarking Membrane Protein Detergent Stability (A) Control membrane proteins FSEC traces in LDAO (green) and 12M (black) which have been ordered from most stable (left) to least stable (right), as determined by their unfolding half-life in LDAO. (B) Bars represent the unfolding half-life for each protein in LDAO. (C) Bars represent the unfolding half-life for each protein in 12M. Bacterial proteins, open bars; Bacterial protein controls, light grey bars; Eukaryotic proteins, black bars. The > sign above BT-3, 9 bars indicate that the half-life stability, although consistent in both 12M and C_12_E_9_ detergents (see Figure S6A) was longer than the time measured to assess stability. See also Figures S5–S7.

As shown in Figure 3A and Figure S5, we can distinguish a noticeable difference in the quality of the FSEC traces as the t_1/2_ in LDAO decreases. All transporters and control membrane proteins with a t_1/2_ longer than 15 min are equally monodisperse in LDAO as they are in 12M. In contrast, all transporters that have a t_1/2_ shorter than 15 min have FSEC traces in LDAO that are noticeably broader than those in 12M, with increasing amounts of aggregation as the t_1/2_ becomes progressively shorter.

To verify at what t_1/2_ the proteins tested here will aggregate with complete exchange into LDAO, all proteins showing similar 12M and LDAO traces were retested using purified protein. Apart from the control with the shortest t_1/2_ of 15 min (NhaA), all transporters and control membrane proteins with a t_1/2_ of 17 min or longer could be exchanged from 12M into LDAO using SEC and concentrated above 10 mg.ml^-1^. As a further test, we submitted the transporters exchanged into LDAO for crystallization trials.

Bacterial transporters BT-5 and BT-8 crystallized in LDAO. While crystals for BT-8 were poor, the crystals for BT-5 improved from 7 Å in 12M, to 2.8 Å and finally to 2.2 Å resolution in LDAO (Figure S2C). By using crystals grown in this detergent, we have recently been able to determine the structure for this transporter (unpublished data) (Table 1B).

Based on this analysis, we estimate four bacterial transporters (open bars) and none of the eukaryotic transporters (black bars) with an unfolding t_1/2_ time of ∼17 min or longer at 40°C can reliably be exchanged into LDAO (Figure 3B). For the control proteins (gray bars), only AmtB, Mhp1, and GlpG-tr, which can be crystallized in either LDAO or 9M detergents, pass this benchmark. A t_1/2_ of ∼17 min or longer at 40°C also fits the stability data obtained for 12M, in which all proteins crystallized (Figure 3C). That is, apart from MT-2 and MT-1 with a t_1/2_ close to this benchmark of 16 min, most proteins have a t_1/2_ = 20 min or longer. The stability of proteins in C_12_E_9_ is also comparable to that measured in 12M; only MT-2, t_1/2_ = 10 min, is clearly unstable in this detergent (Figure S6A). In addition to 12M, C_12_E_9_, and LDAO detergents, we consistently find that proteins with a t_1/2_ less than 15 min in 10M and 9M always aggregate upon exchange from 12M into these detergents (data not shown). Thus, we estimate that the majority of bacterial transporters, with a t_1/2_ of 17 min or longer at 40°C, are also sufficiently stable to be exchanged from 12M into 10M (9/9) and 9M (6/9) (Figures S6B and S6C). Half of the eukaryotic transporters tested are suitable for exchange into 10M (5/8) or 9M (4/8) (Figures S6B and S6C). Of the few eukaryotic transporters stable in 10M and 9M detergents, all are plant transporters, while none are mammalian transporters. This analysis is also consistent with our greater success at obtaining better diffracting crystals for plant transporters (Table 1B).

To assess if sensitivity toward lipid loss or incompatibility could be the reason for the poor detergent stability of the mammalian transporters, we added the lipid mixture PC: PE: PG into the SEC buffer containing 12M as previously described (Long et al., 2005). MT-2 and MT-3 transporters were both more stable in 12M with lipid addition, although no improvement was seen for MT-1 and MT-4 (data not shown). For MT-2 which showed the most improved stability, with an increase in t_1/2_ from 16 to 58 min, crystals grew with a different morphology and diffract X-rays at much higher resolution: 6 Å in the presence of lipid compared to 15 Å without (Figure S7).

### Membrane Protein Stability Is Predominantly Intrinsic Not Detergent Specific

To establish if membrane proteins that are comparatively more stable in one detergent are also more stable in other types of detergents, we compared the membrane protein unfolding rates of the proteins included in this study against one another.

**Figure 4:**
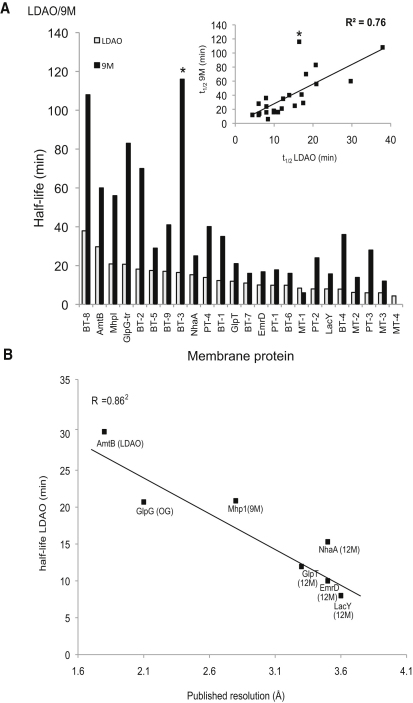
Membrane Protein Stability Is Predominantly Intrinsic and Is Related to Its Propensity to Form Well-Ordered Crystals (A) Bars represent the unfolding half-life for each protein in 9M (filled) and plotted against that measured in LDAO (nonfilled); unfolding rates for LDAO were plotted from the highest to lowest (left to right). Inset is a linear curve indicating the average stability difference between LDAO and NM. Asterisk for protein BT-3 indicates that we considered this difference as an outlier and, as such, was not included in calculation of the correlation coefficient as displayed in inset. (B) Membrane protein stability, as judged by unfolding rates in the detergent LDAO, correlates to the published resolution of control membrane proteins. The detergent used for crystallization is listed in brackets besides each protein. See also Figure S8.

As shown in Figure S8A, there is a reasonably good correlation between the t_1/2_ in 9M and 10M (R^2^ = 0.76). A similar correlation is observed for the t_1/2_ of the membrane proteins in 12M and C_12_E_9_ (R^2^ = 0.86) Figure S8B, and also for 9M compared to LDAO (R^2^ = 0.76) (Figure 4A). However, there are a number of transporters that do not conform to this trend. For example, Mhp1 is more stable in 9M, t_1/2_ = 56 min, than what would expect from the half-life in 12M, t_1/2_ = 43, presumably because it particularly favors this detergent; indeed, 9M was used for crystallization of Mhp1 (Weyand et al., 2008). Overall, however, these findings strongly indicate that membrane protein stability is predominantly intrinsic rather than detergent specific.

So far, we have obtained well-diffracting crystals for BT-2, BT-5, and BT-9 (Table 1B). With the exception of BT-8, these are the only transporters stable in LDAO, Figure 3B. In light of this observation we focused on improving the diffraction from BT-8. After careful optimization, BT-8 crystals were obtained that diffract up to 3.3 Å in 12M (Table 1B; Figure S2C). If we compare the stability of all the peptide transporters screened here, namely, BT-6, BT-7, BT-8, and BT-9, it is clear that BT-8 and BT-9 are more stable overall (Figure S9). We suggest that this approach can aid in the selection of orthologs best suited for structural studies. Even with unrelated transporters we see a stability trend, only the bacterial transporters stable in LDAO (BT-2, BT-5, BT-8, BT-9) have yielded crystals that diffract X-rays from medium to high resolution (Figure 3B).

A large-scale structure analysis of globular proteins concluded that crystallization propensity is not substantially influenced by thermodynamic stability. The best correlation is the frequency of well-ordered surface epitopes capable of mediating protein-protein interactions (Price et al., 2009). In agreement with this analysis we also find no correlation between the stability of the membrane protein in 12M and the propensity of the protein to crystallize. Transporters we have been unable to crystallize are, on average, as stable as those that do crystallize (data not shown). However, if membrane proteins can be crystallized in 12M, our findings indicate that the better the stability the greater the likelihood of optimizing crystals to diffract X-rays to higher resolution. For the control proteins, this is also the case. This correlation appeared in all tested detergents but again is clearest in the detergent LDAO (Figure 4B).

### Conclusion

In total, 25 pro- and eukaryotic membrane proteins consisting of channels, enzymes, and primary and secondary active transporters were crystallized from protein expressed as GFP fusions in *E. coli* or *S. cerevisiae*. To overcome the greatest stumbling block for structure determination, that of crystal optimization, we have measured the stability of 24 membrane proteins in various detergents using a fluorescent-based unfolding assay.

Our findings indicate that membrane proteins with an unfolding rate longer than approximately 17 min at 40°C are sufficiently stable for crystallization trials in that detergent. Because we find that membrane protein stability is an intrinsic property, rather than one conferred by the detergent, proteins stable in harsher detergents, like LDAO, are likely to be more stable in a more diverse range of other detergents. These proteins are not only more likely to form crystals in 12M that diffract X-rays from 3 to 3.6 Å, but also to higher resolution in a small micelle-sized detergents. Using this information, we have been able to identify target homologs for crystallization trials to speed up structural determination. We have also been able to confirm a relationship between the monodispersity of a membrane protein in crude LDAO solubilized membranes with its purified stability. This further validates the use of the FSEC method for rapidly screening stable homologs prior to purification.

These findings do not mean those membrane proteins that do not pass this stability benchmark cannot be optimized in 12M to yield well-diffracting crystals over time, e.g., NhaA. However, we suggest that membrane proteins that pass this benchmark optimize faster because proteins that are this stable have a larger “crystallization space.” Most likely membrane proteins that have large hydrophilic domains do not need to be as stable because extensive crystal contacts can already be formed in large micelle-sized detergents. This is also true for scaffold approaches that increase the surface area for crystal contacts in a mild detergent, e.g., T4-lysozyme GPCR fusions (Rosenbaum et al., 2007) and/or more general membrane protein FAB/Fv monoclonal fragment complexes (Dutzler et al., 2003). Still, even in both these cases, we argue that crystal optimization is likely to be more fruitful if efforts are placed on the homolog that is the most stable. If no naturally stable variants are found, then effort can be placed on the identification of mutants or ligands that stabilize the protein. In this regard it is important for future stabilization strategies, in particular of mammalian homologs, that we have verified that stability is predominantly intrinsic as it further rationalizes the general use of such approaches.

In summary, by using the stability benchmarks outlined here, it is possible to identify targets and optimize constructs that are more likely to yield well diffracting crystals; by doing so we can increase the rate at which new high-resolution eukaryotic and prokaryotic membrane protein structures are solved.

## Experimental Procedures

### Yeast and *E. coli* Genetic Manipulations

Eukaryotic membrane proteins were amplified from their respective cDNA (either obtained from collaborators or purchased from imaGenes http://www.imagenes-bio.de/), and bacterial/archeal membrane proteins from genomic DNA with exception of *E. coli* clones which were obtained from an already constructed *E. coli* GFP-fusion library (Daley et al., 2005). Cloning into *S. cerevisiae* primers contained a 18 bp gene specific region and a 30 bp homologous region on the forward 5′-TCG ACG GAT TCT AGA ACT AGT GGA TCC CCC-3′ and reverse primer 5′-AAA TTG ACC TTG AAA ATA TAA ATT TTC CCC-3′. For cloning into *E. coli* vector pWaldo GFPe, primers contained a 21 bp gene specific region, and a 5′-GCGCCCTCGAG-3′ overhang on the forward primer for XhoI digestion and a 5′-CGCGCGGAATCC-3′ overhang on the reverse primer for EcoRI digestion (Drew et al., 2006). PCR product for eukaryotic genes and SmaI linearized pDDGFP-2 (Newstead et al., 2007) were transformed into the FGY217 strain *(MATa*, *ura3-52*, *lys2Δ201*, *pep4Δ*) (Kota et al., 2007). Transformants were selected on -Ura plates and positive clones initially confirmed by colony PCR and/or whole-cell GFP fluorescence. For cloning into *E. coli* XhoI/EcoRI digested bacterial or archeal PCR products were transformed with cut vector into cloning cells and selected on plates containing 50 μg/ml Kanamycin.

### Overexpression and Purification of Membrane Proteins Using GFP-Based *E. coli* and *S. cerevisiae* Pipelines

Detailed step-by-step protocols describe our GFP-based overexpression and purification pipeline in *E. coli* (Drew et al., 2006) and in *S. cerevisiae* (Drew et al., 2008). In brief, membrane protein-GFP-His_8_ fusions were selected based on fluorescence counts from 10 or 1 ml cultures corresponding to more than 1 mg per liter. Membranes were isolated from 2 liter cultures, resuspended in 10 ml of buffer, and 0.2 ml was used for solubilization in 1% v/v with either of the detergents C_12_E_9_, 12M, 10M, 9M, or LDAO and analyzed for monodispersity by FSEC. Monodisperse fusions in 12M were selected for large-scale culturing (10–15 liters) and purified by IMAC in 1 × PBS buffer containing 0.1% 12M as detailed in referenced protocols. Fusions were cleaved overnight with equimolar His_6_-TEV protease during dialysis into 3 liter of crystallization buffer containing 20 mM Tris-HCl (pH 7.5), 0.1M NaCl, 0.03% 12M. Digested material was passed through a 5 ml His-Trap column (GE Healthcare), and the flow through containing the target protein collected. Membrane protein was concentrated, and loaded onto a Superdex 200 10/30 size-exclusion column at 0.4 ml/min in crystallization buffer in the presence or absence of a lipid mixture (3PC: 1PE: 1PG, Avanti Polar Lipids). The monodisperse protein peak was collected and concentrated with either 50K or preferably 100K MWCO (Millipore) concentrators to ∼8–20 mg/ml.

### Generalized Crystallization and Data Collection Strategy

Screening was carried out using the targeted sparse-matrix MemGold, MemSys, and MemStart screens (Molecular Dimensions Ltd) using the Mosquito crystallization robot (TTP LabTech) in 96-well plates (MRC, Germany). All screens were set up with 200 nl drop sizes and at 19°C and 4°C. Crystal optimization was carried out manually using hanging drop plates in a 24-well setup using drop sizes of 1 μl and using 96-well detergent and additive screens (Hampton, Aliso Veijo, CA). Diffraction screening and data collection was carried out at various synchrotron beamlines at the Diamond Light Source (IO2, IO3, IO4, I24) and the ESRF (ID14eh4, ID29, ID23eh1, and ID23eh2). Data were processed and scaled using the HKL suite of programs (Otwinowski and Minor, 1997).

### 96-Well Fluorescent Based CPM Thermostability Assay

The thermostability assay was carried out essentially as described by Stevens and co-workers (Alexandrov et al., 2008; Roth et al., 2008). One microliter of purified membrane protein at ∼10 mg/ml was added to 150 μl of buffer containing 20 mM Tris-HCl (pH 7.5), 0.1 M NaCl, and detergent at three times CMC in a 96-well black Nunc plate. CPM dye at 4 mg/ml in DMSO was diluted 100-fold into buffer containing 20 mM Tris-HCl (pH 7.5), 0.1 M NaCl, 0.03% DDM, and warmed to room temperature. Three microliters of dye at 40 μg/ml was added, clear plate cover set in place, and within 5 min from protein addition fluorescence emission was measured at 463 nm (excitation 387 nm) on the SpectraMax^2e^ plate reader (Molecular Devices) at 40°C. Recordings were measured every 5 min for 3 hr with 15 s shaking interval between each reading. The fraction of folded protein at each time point was calculated by the quotient of raw fluorescence measured at each time point divided by the maximal fluorescence measured for the detergent series. A single exponential decay curve was plotted using GraphPad Prism software (San Diego, CA).

## Figures and Tables

**Table 1:** Summary of Crystallization and Corresponding X-ray Diffraction Obtained for Prokaryotic and Eukaryotic Transporters.

(A) The Summary of the Expression, Purification, and Crystallization Success Rates of the 140 Eukaryotic and 52 Prokaryotic Transporters Screened from 12 Different Transporter Families


(B) Summary of the X-ray Diffraction Obtained from Transporter Crystals and Control Membrane Proteins Screened in (A)


Proteins are designated as BT, bacterial transport protein; AT, archeal transport protein; MT, mammalian transport protein; PT, plant transport protein. Diffraction limits were binned into either >3.5, 4–8, or <8 Å due to anisotropic diffraction (>3.5 Å was restricted to crystals for which a complete data set could be collected and scaled to this resolution or higher). Left brackets indicate initial resolution of screened crystals, no brackets indicates the resolution obtained after further testing using standard crystallographic methods and right brackets, with resolution in italics, is the final resolution poststability analysis. All proteins were crystallized in 12M except the final resolution as noted for BT-5 (LDAO), AmtB (LDAO), GlpG (10M), and Mhp1 (9M). The dashed line (-) means we were unable to discern clear X-ray diffraction. Note: diffraction shown here is for controls GlpT and GlpG is for the native sequence; however, published structures contain and C- and N-terminal truncations respectively (Wang et al., 2003, 2006).

## Supplemental Information

Document S1. Nine Figures
