# Supplementary material for: Benchmarking Membrane Protein Detergent Stability for Improving Throughput of High-Resolution X-ray Structures
Source: Structure. 2011 Jan 12;19(1):17–25. doi: 10.1016/j.str.2010.12.001 (PMC3111809; doi:10.1016/j.str.2010.12.001)
Supplement: Document S1. Nine Figures [file mmc1.pdf]

Structure *19*

Supplemental Information

Benchmarking Membrane Protein Detergent Stability for Improving Throughput of High-Resolution X-ray Structures

Yo Sonoda, Simon Newstead, Nien-Jen Hu, Yilmaz Alguel, Emmanuel Nji, Konstantinos Beis, Shoko Yashiro, Chiara Lee, James Leung, Alexander D. Cameron, Bernadette Byrne, So Iwata, and David Drew

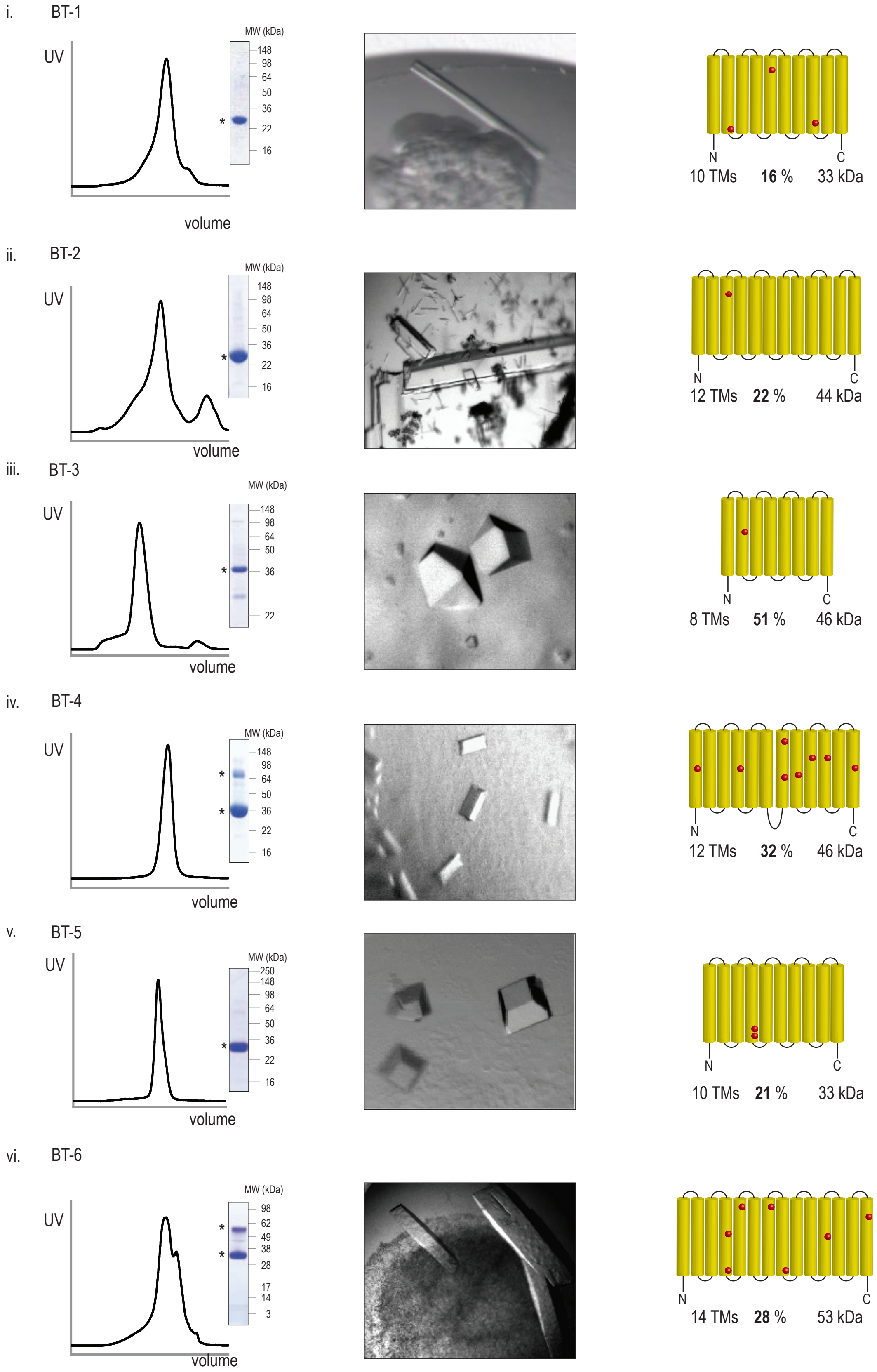

Figure SI 1a(i-vi),related to Table 1, Bacterial transporter SEC,crystallization, and TMHMM topology prediction

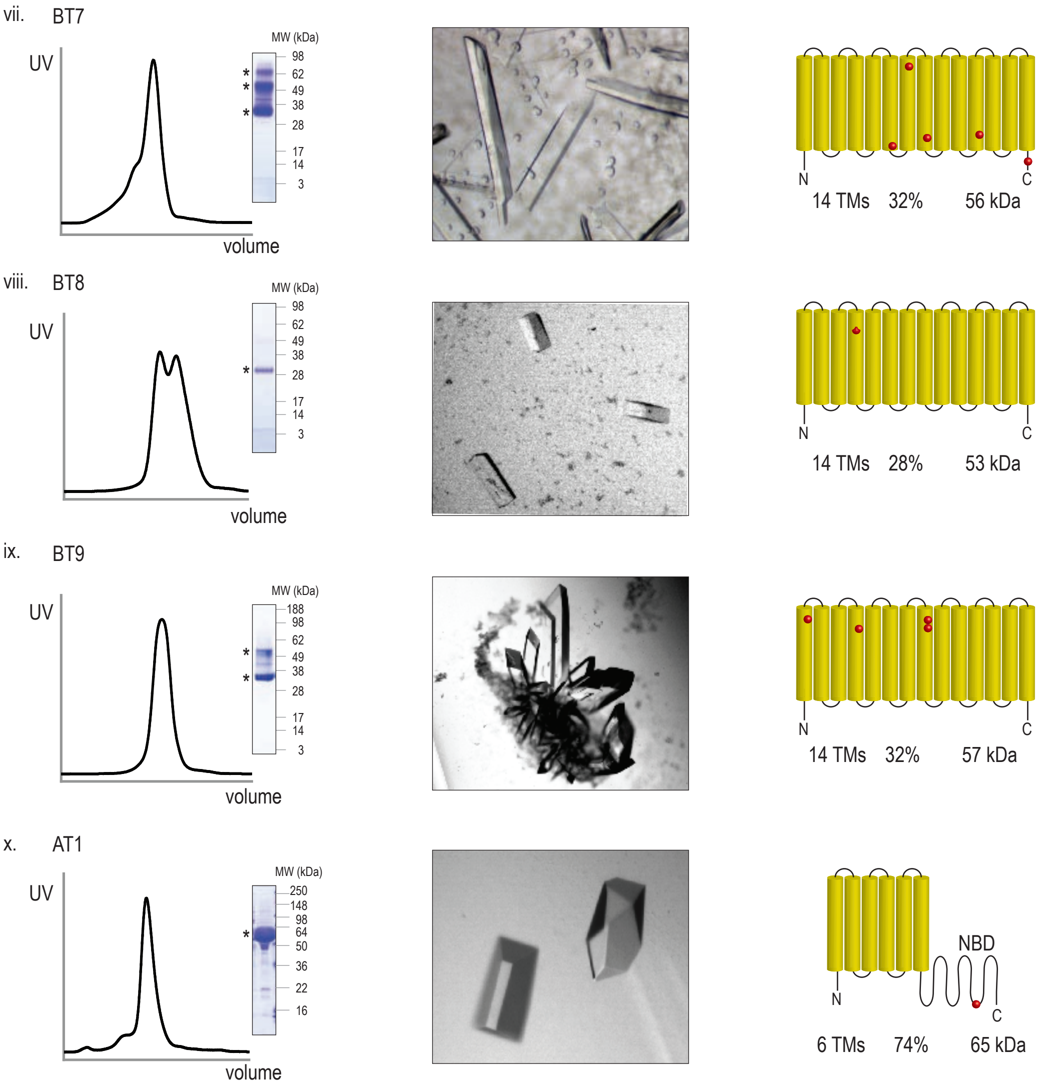

Figure SI 1a(vii-x),related to Table 1, Bacterial and archeal transporter SEC, crystallization and TMHMM topology prediction

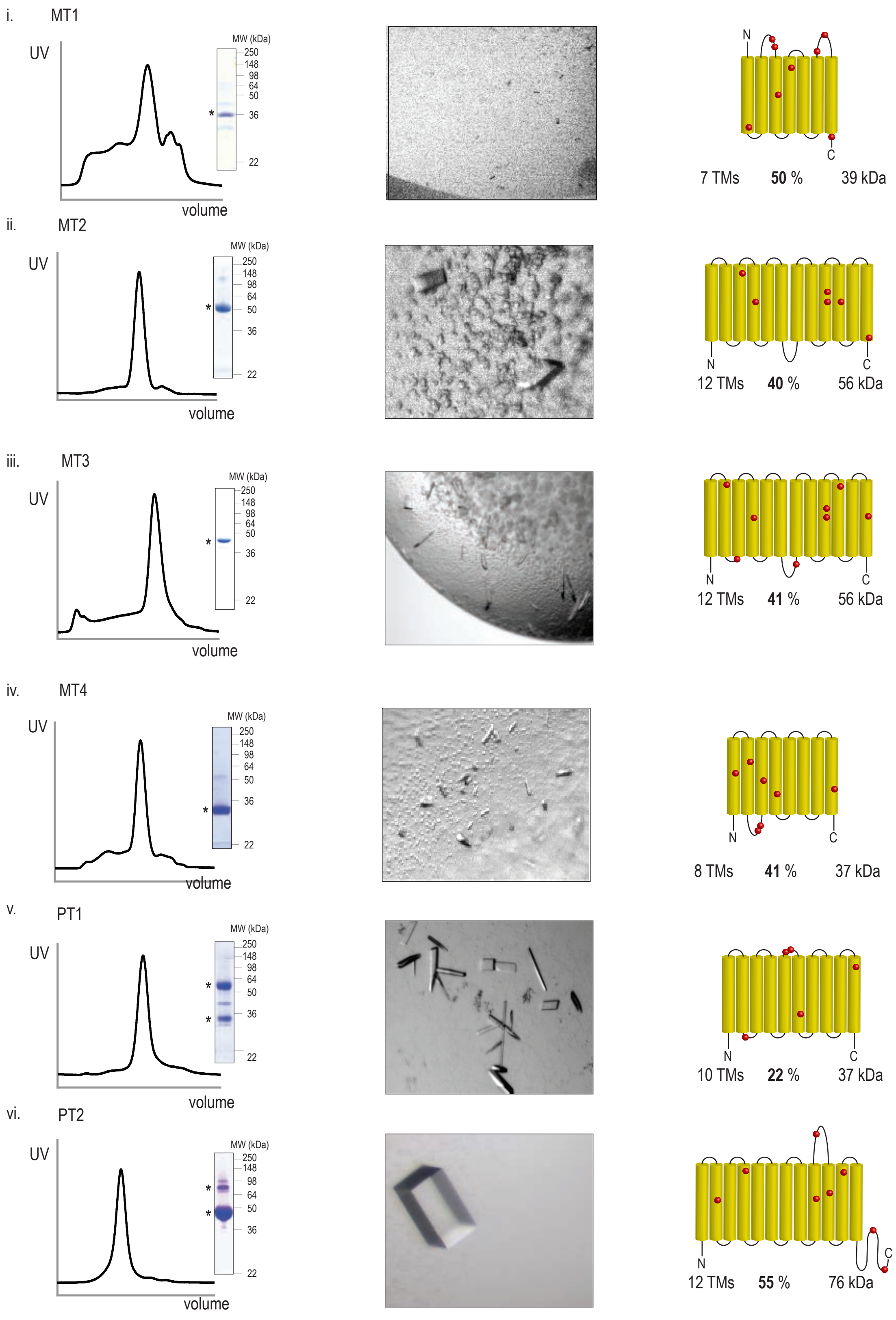

Figure SI 1b(i-vi),related to Table 1, Eukaryotic transporter SEC,crystallization and TMHMM topology prediction

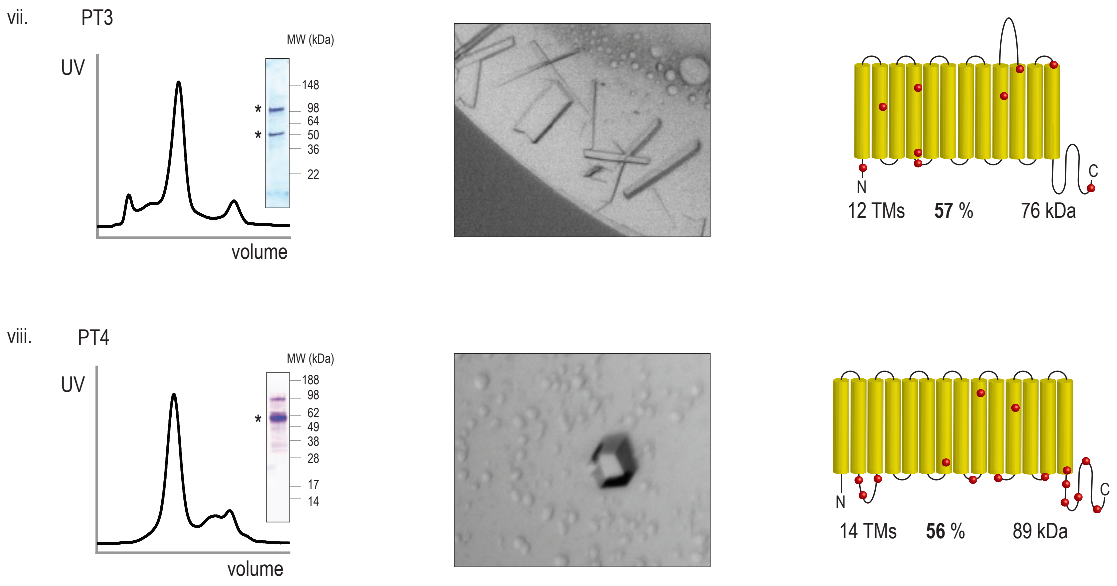

Figure SI 1b(vii-viii),related to Table 1, Eukaryotic transporter SEC,crystallization and TMHMM topology prediction

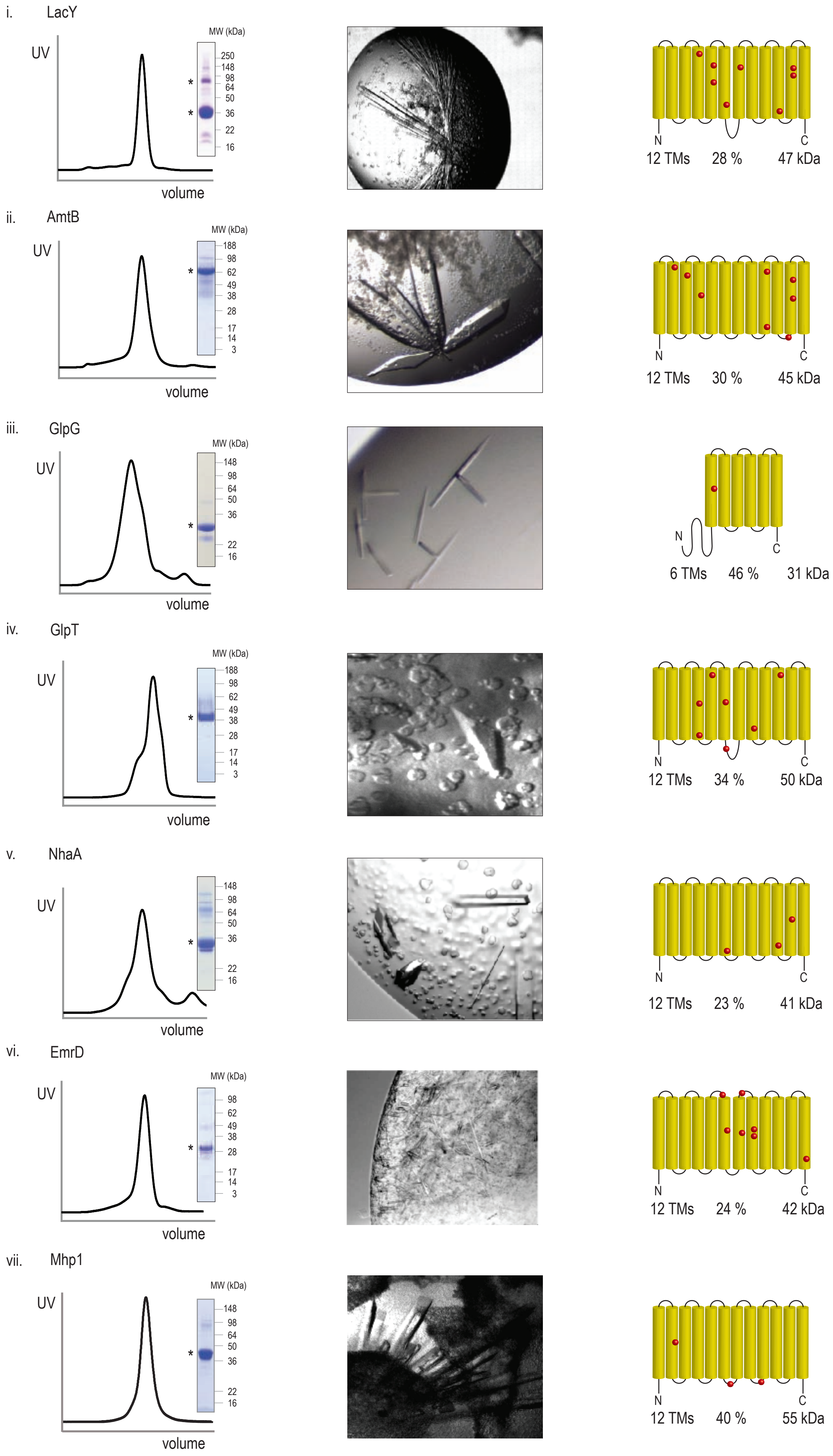

Figure SI 1c(i-vii),related to Table 1, Control membrane protein SEC,crystallization and TMHMM topology prediction

Figure 1, related to Figure 1. Topology and SEC analysis of membrane proteins that crystallize in the detergent 12M

SEC traces in 12M are shown, left panel, with the SDS-PAGE analysis of each protein shown (asterix's indicate the target protein bands). Crystal pictures taken on a standard light microscope, middle panel, captured and rendered using Adobe^{TM} software. Topology models of each protein as predicted by TMHMM, with helices depicted as yellow rods and the position of the cysteines illustrated with red spheres. The numbers represent the number of TMs, the fraction of non-TM residues as inferred from the topology and the predicted molecular weight of the protein (kDa). **a**. Bacterial transporters: i. BT-1, ii BT-2, iii. BT-3, iv BT-4, v. BT-5, vi. BT-6, vii. BT-7, viii. BT-8, ix. BT-9, x. AT-1. **b**. Eukaryotic transporters: i. MT-1, ii. MT-2, iii. MT-3, iv. MT-4, v. PT-1, vi. PT-2, vii. PT-3, viii. PT-4. **c**. Bacterial control membrane proteins. i. LacY, ii. AmtB, iii. GlpG, iv. GlpT, v. NhaA, vi. EmrD, vii. Mhp1. Note, because AT-1 does not harbour any TM-embedded cysteines it was excluded from the stability analysis.

i.

PT-2

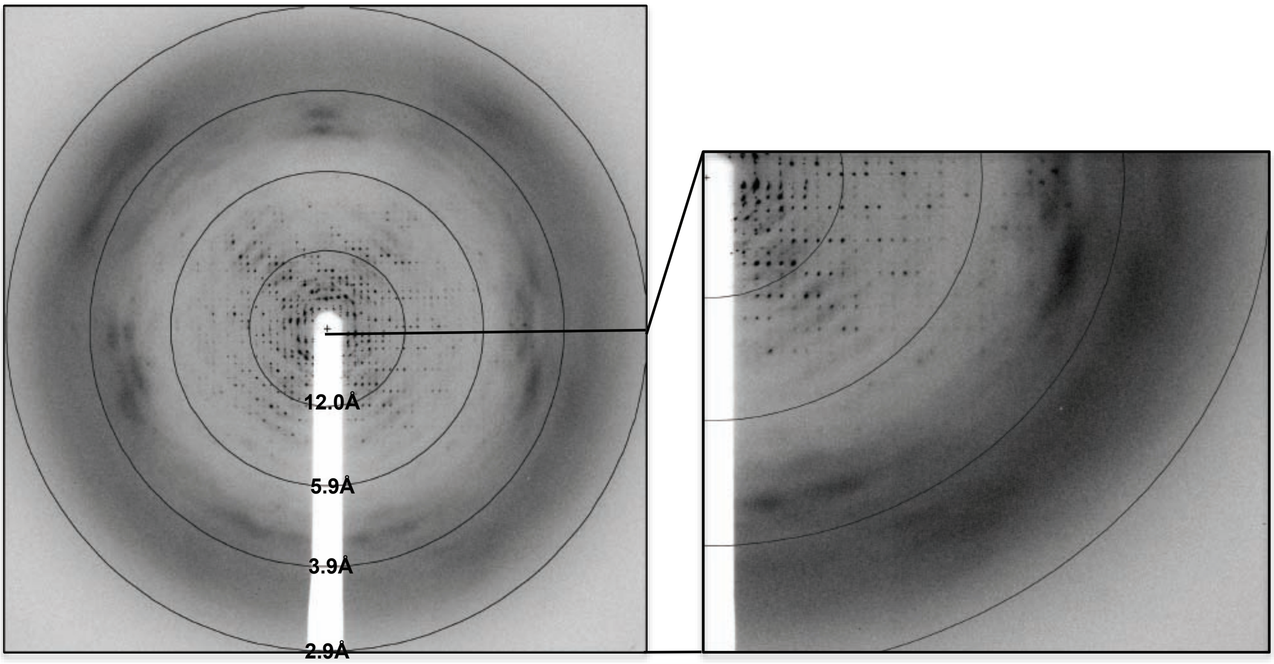

ii.

BT-2

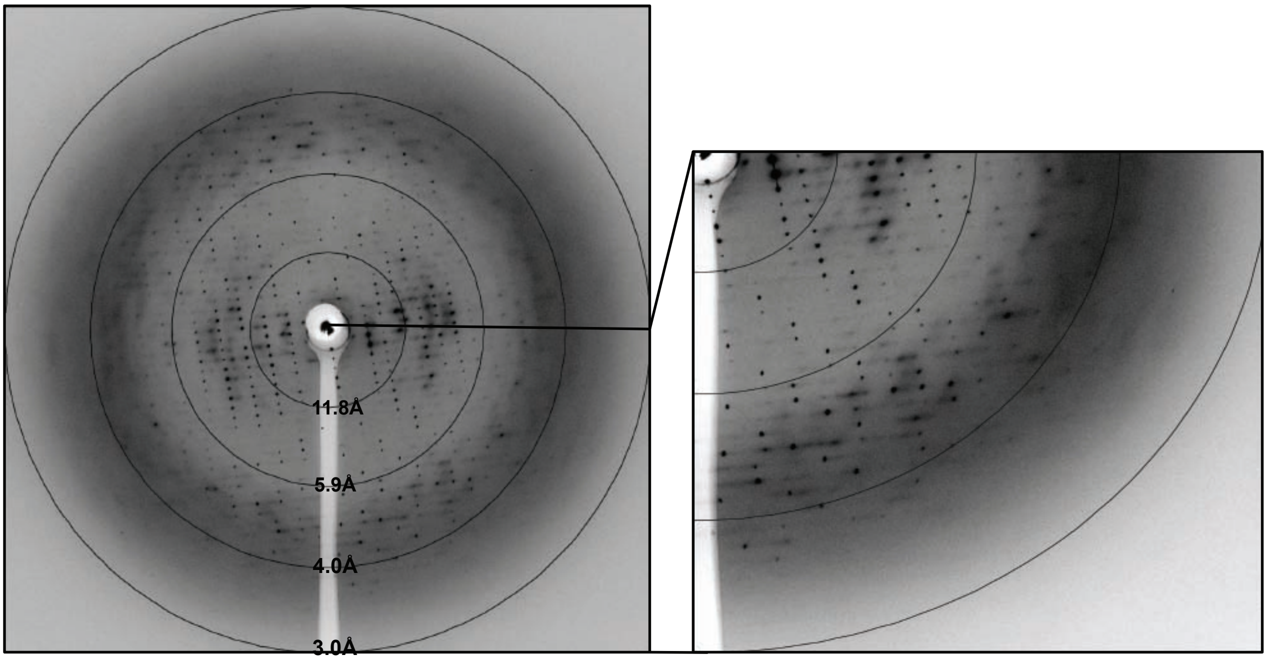

Figure SI 2a(i-ii),related to Table 1,
X-ray diffraction of PT-2 and BT-2

iii.

BT-3

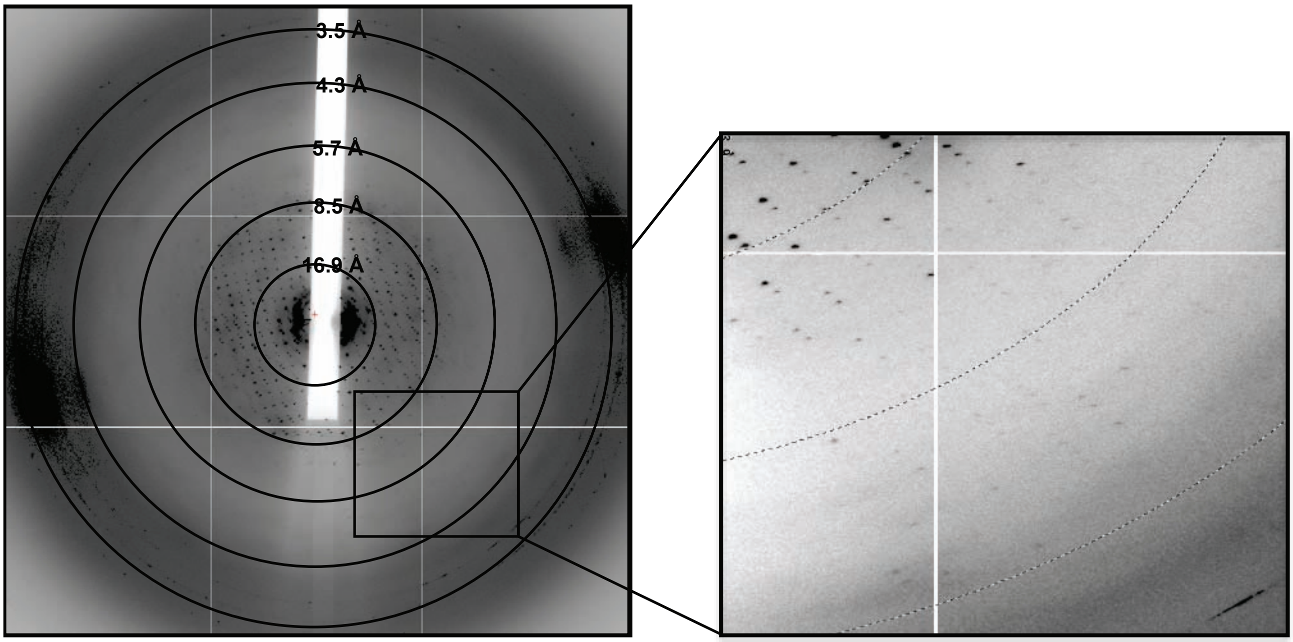

Figure SI 2a(iii),related to Table 1,
X-ray diffraction of BT-3

i.

AmtB

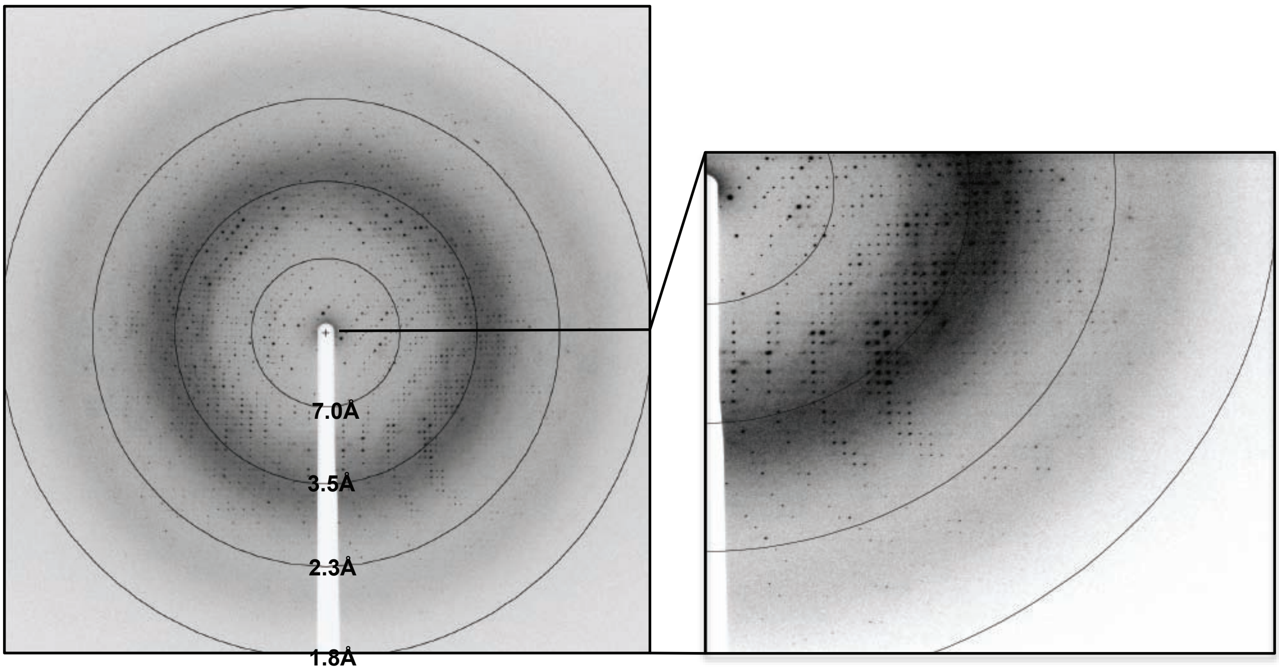

ii.

Mhp1

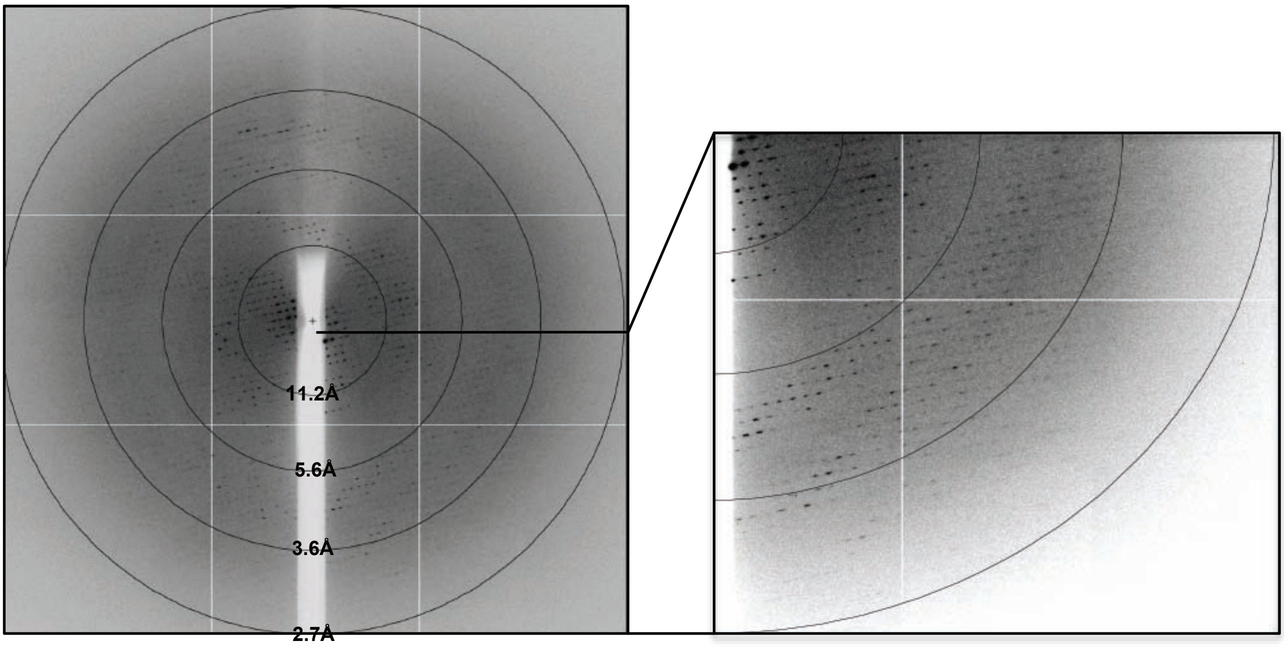

Figure SI 2b(i-ii), related to Table 1, X-ray diffraction of control membrane proteins AmtB and Mhp1

iii.

GlpG

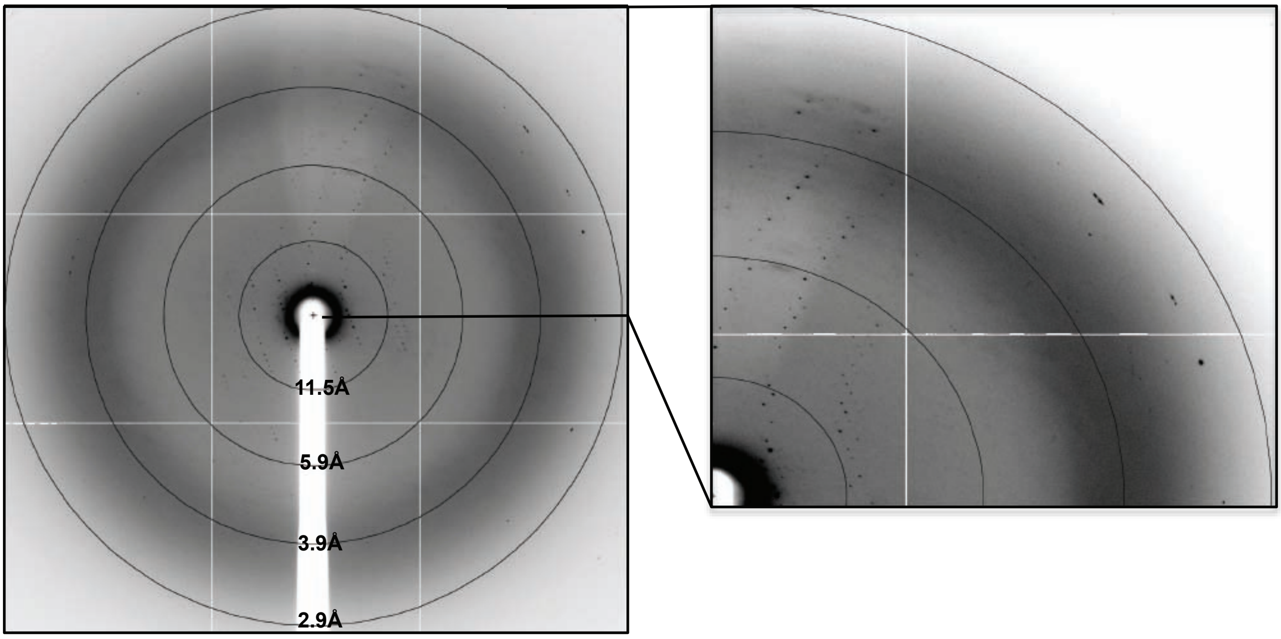

iv.

NhaA

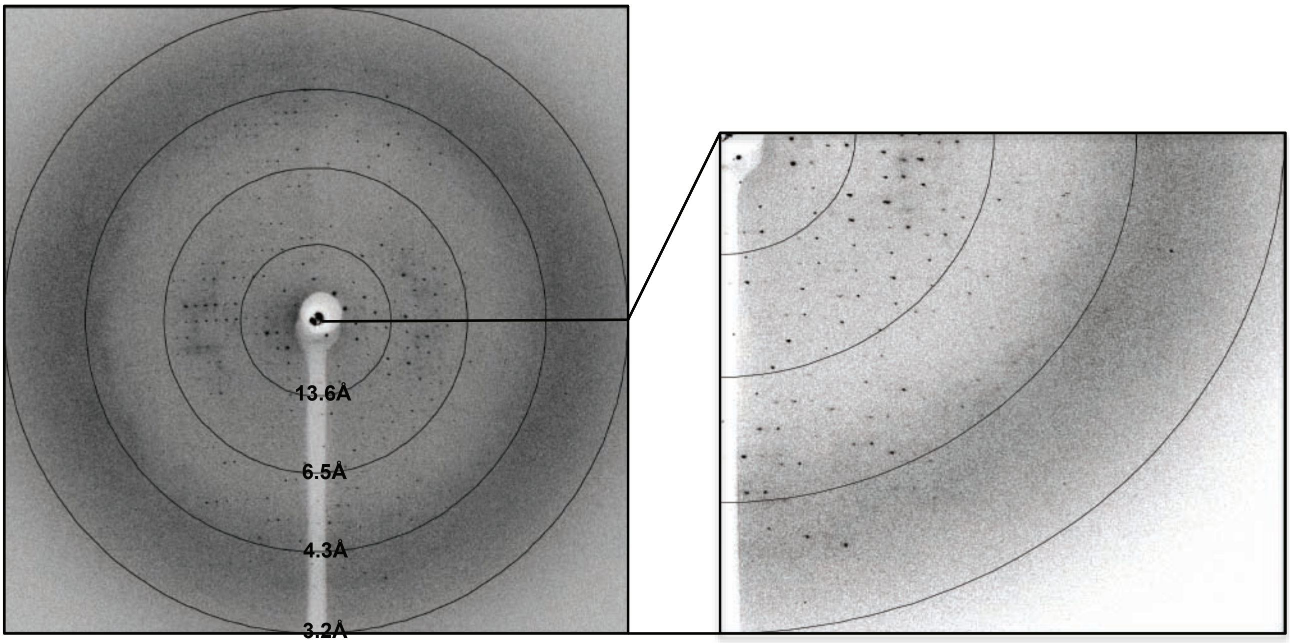

Figure SI 2b(iii-iv), related to Table 1, X-ray diffraction of control membrane proteins GlpG and NhaA

i. **BT-9**
$PepT_{so}$

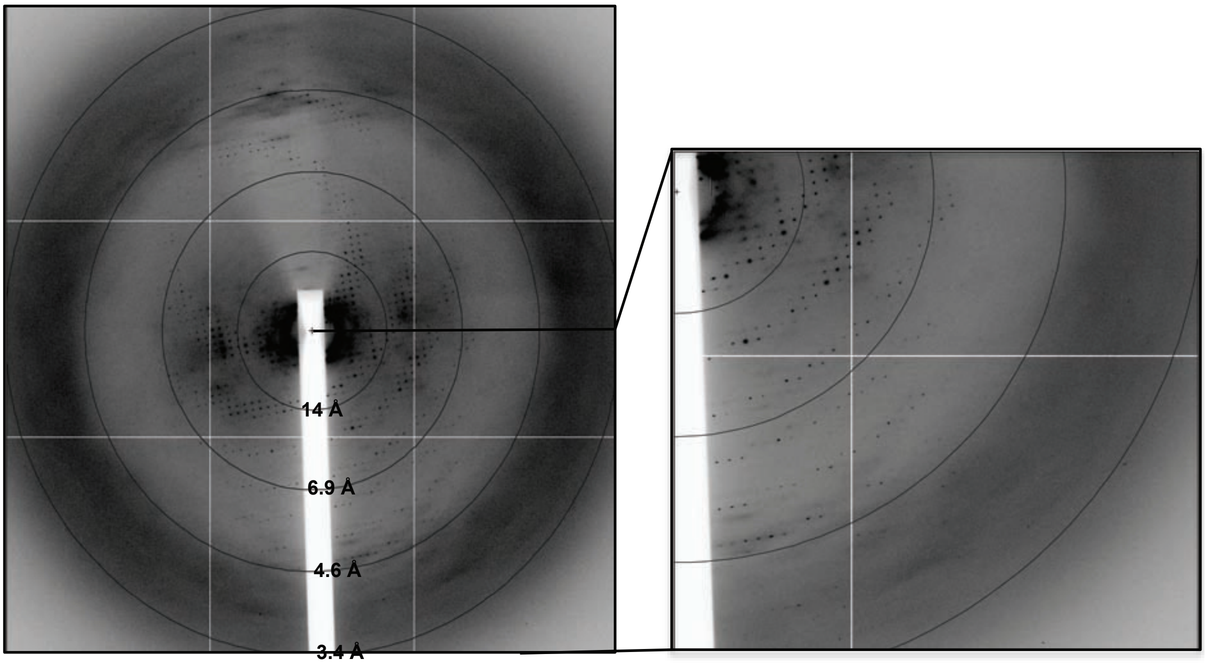

ii. **BT-5**

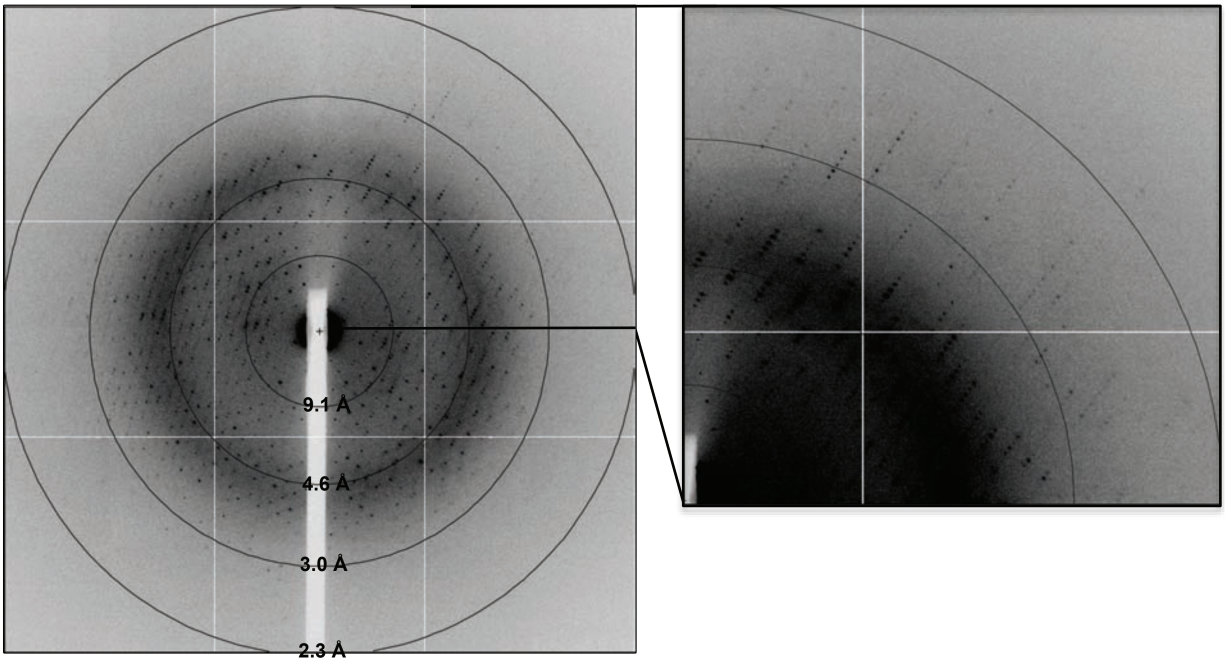

Figure SI 2c(i-ii), related to Table 1, X-ray diffraction of BT-9 and BT-5

iii. **BT-8**

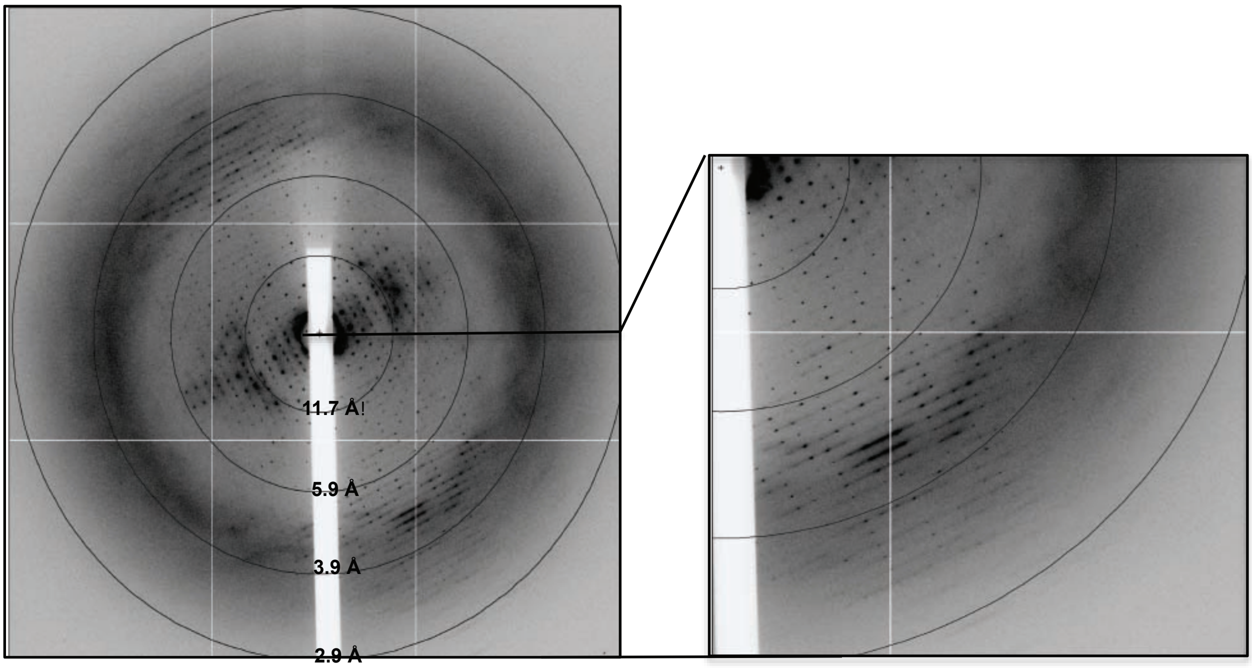

Figure SI 2c(iii), related to Table 1,
X-ray diffraction of BT-8

Figure 2, related to Figure 1. X-ray diffraction images for bacterial and eukaryotic transporters

a. Proteins for which crystals could be grown to 4-Å or above in 12M after optimization. i. PT-2, ii. BT-2, iii. BT-3. **b**. Diffraction of previously crystallized control proteins. i. AmtB (LDAO), ii. Mhp1 (9M), iii. GlpG-tr (10M), NhaA (12M) C. Improved diffraction of the peptide transporter using material produced from the GFP-based pipeline BT-9, $PepT_{So}$ (i), using the stability analysis for exchanging BT-5 into the detergent LDAO (ii) and for the identification of BT-8 as a stable homologue for further optimization (iii). Resolution limits are shown as concentric circles with numbers representing the resolution in Angstroms.

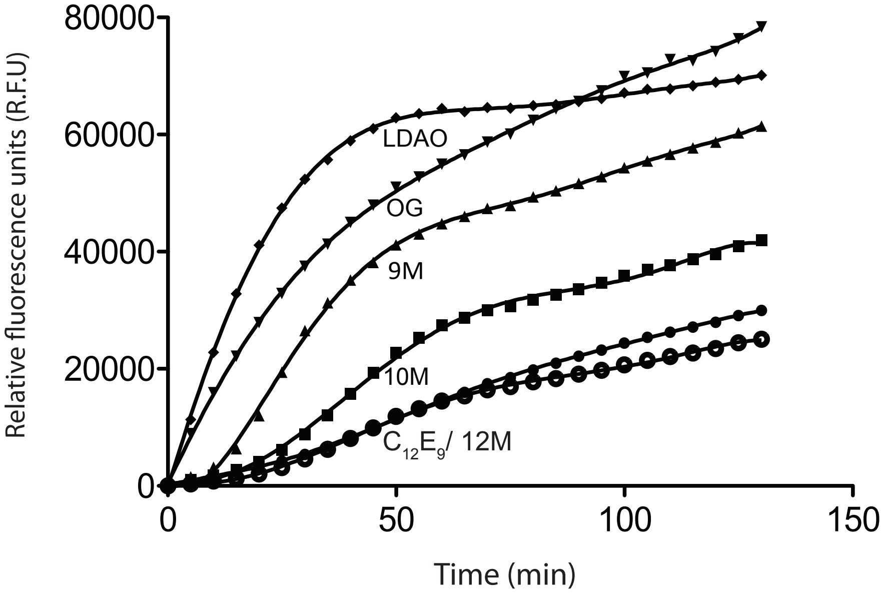

Figure SI 3, related to Figure 2a, raw data from CPM assay

Figure 3, related to Figure 2. Raw data generated by the CPM assay for the bacterial transporter BT-4

Typical data generated by the CPM assay, as shown here for the bacterial transporter BT-4, at 40°C for 130 min in each of the detergents used for analysis as indicated.

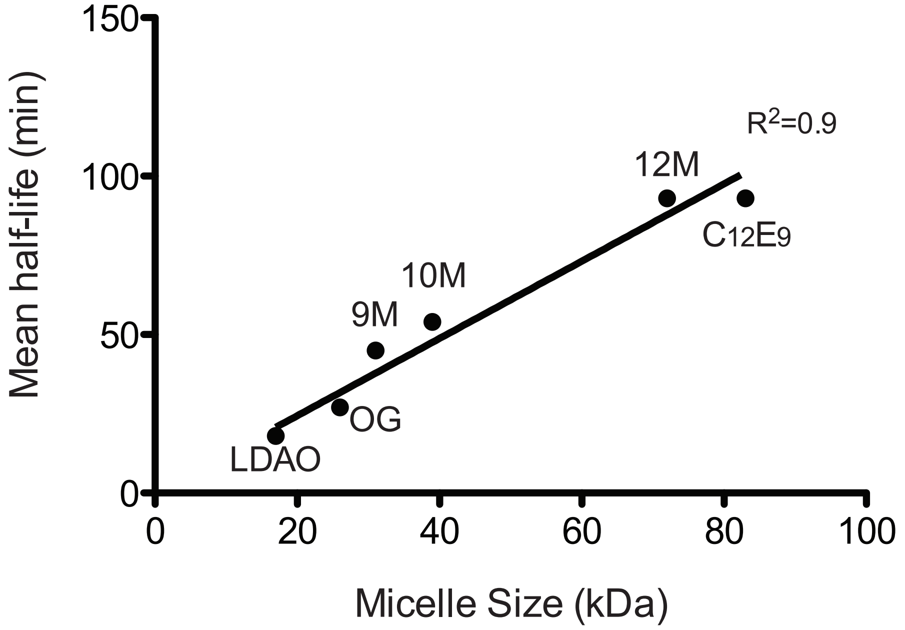

Figure SI 4,related to Figure 2b, relationship between membrane protein detergent stability and micelle size of the detergent

Figure 4, related to Figure 2. The relationship between mean prokaryotic membrane protein stability versus the size of the detergent micelle

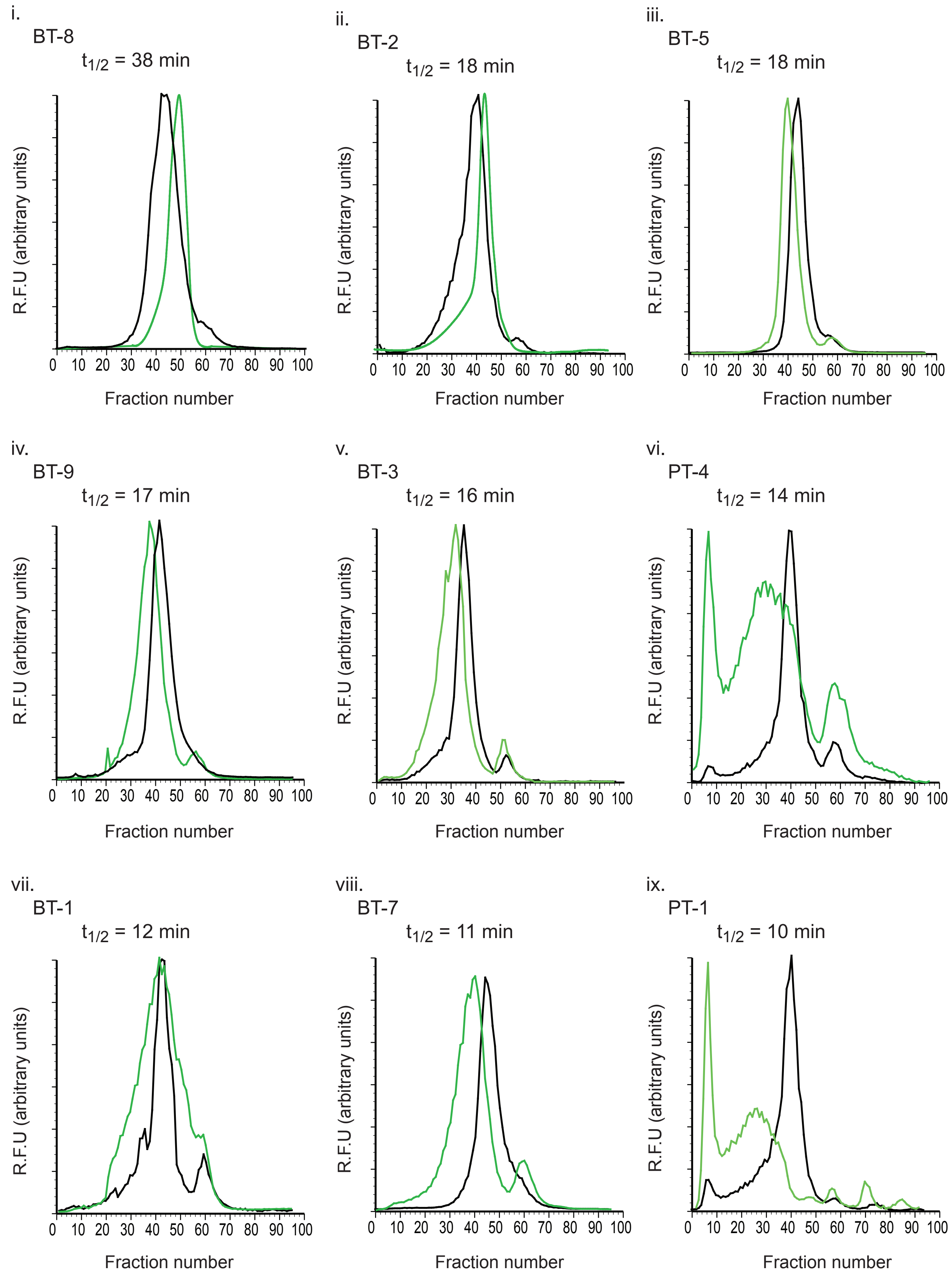

Figure SI 5(i-ix), related to Fig. 3a, comparing monodsipersity profiles to unfolding rates in LDAO.

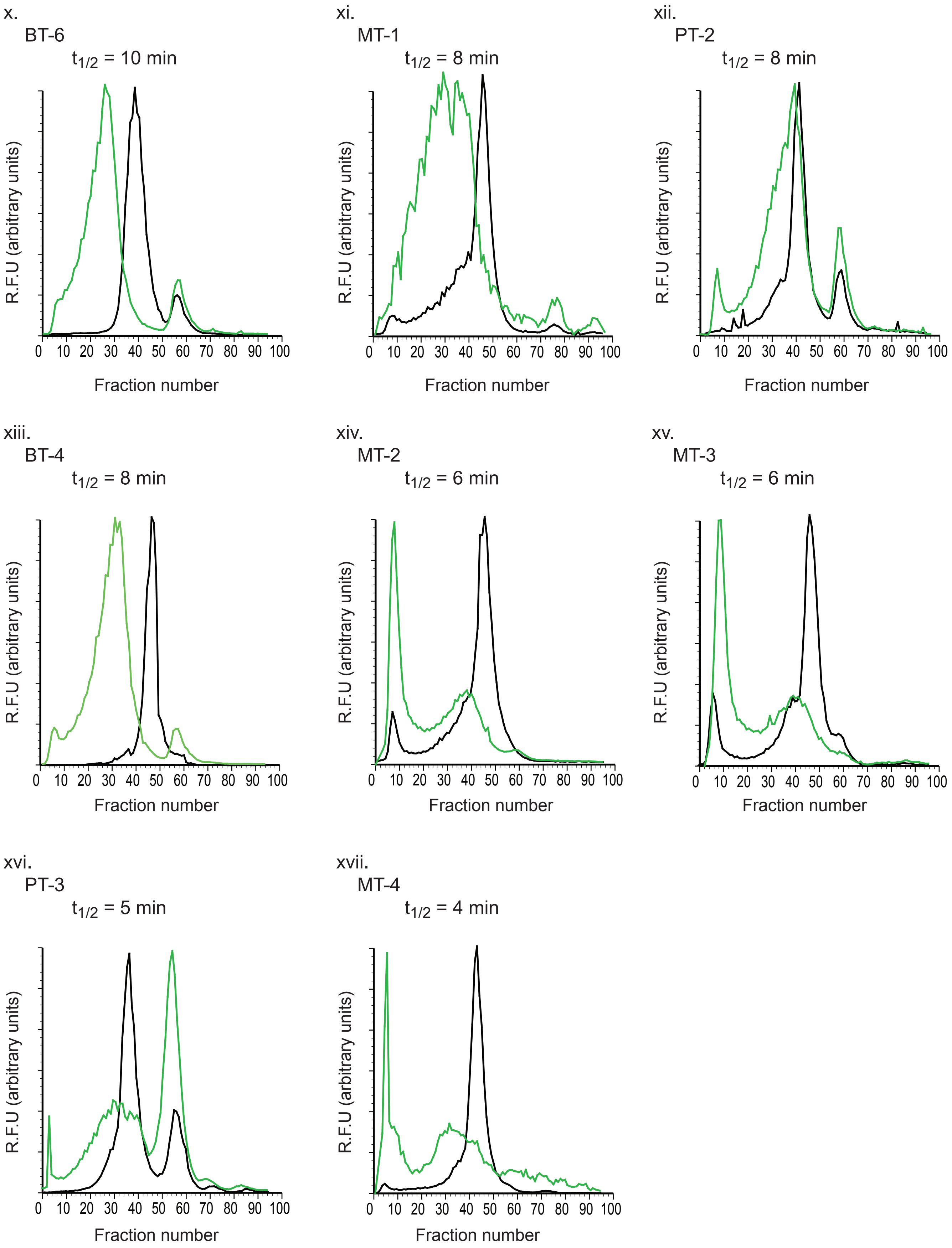

Figure SI 5(x-xvii), related to Fig. 3a, comparing monodsipersity profiles to unfolding rates in LDAO.

Figure 5, related to Figure 3. Benchmarking the stability estimate from the CPM assay by comparing elution profiles of proteins in 12M to the profiles in LDAO Membrane proteins FSEC traces in LDAO (green) and 12M (black) which have been ordered from most stable (left) to least stable (right), as determined by their unfolding half-life in LDAO: i. BT-8, ii. BT-2, iii. BT-5, iv. BT-9 ($PepT_{So}$), v. BT-3, vi. PT-4, vii. BT-6, viii. BT-7, ix. PT-1, x. BT-6, xi. MT-1, xii. PT-2, xiii. BT-4, xiv. MT-2, xv. MT-3, xvi. PT-3, xvii. MT-4. Green represents the FSEC trace in LDAO and black in 12M.

a.

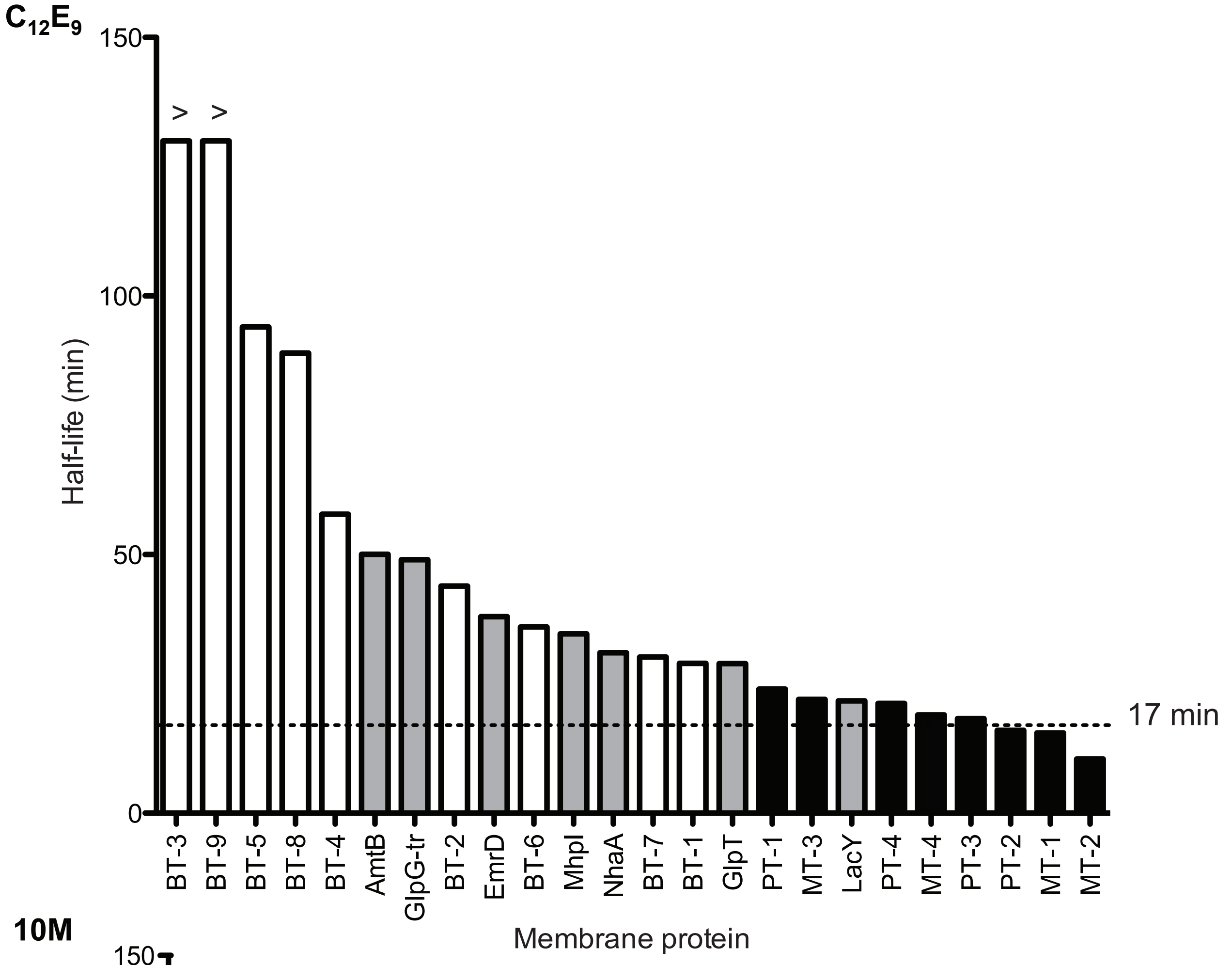

b.

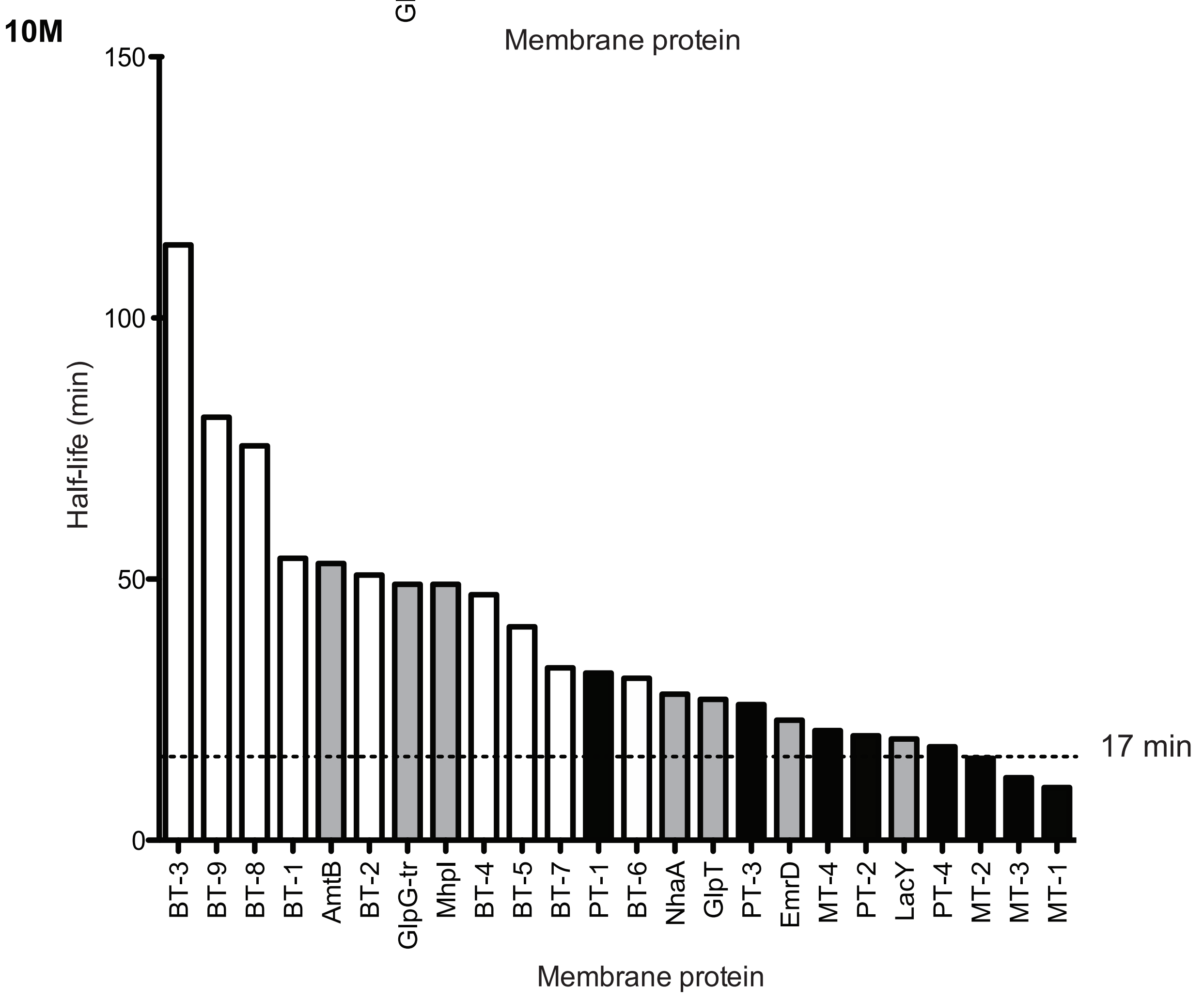

Figure SI 6a and b, related to Figure 3a and b, stability comparison of membrane proteins in the detergent $C_{12}E_9$ and 10M

c.

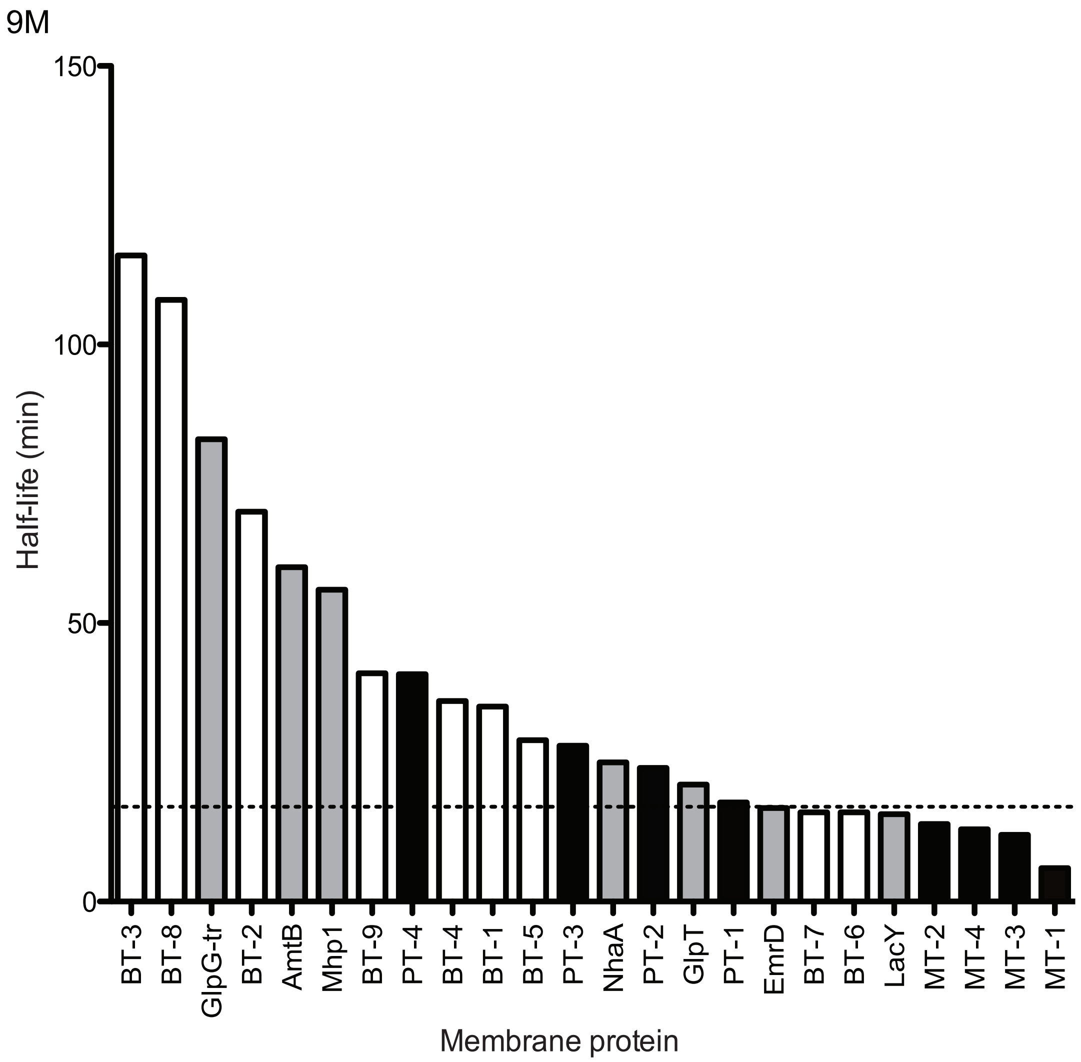

Figure SI 6c, related to Figure 3a and b, stability comparison of membrane proteins in the detergent 9M

Figure 6, related to Figure 3. Comparing the purified stability of 24 membrane proteins in commonly used crystallisation detergents

Bars represent the half-life for each protein calculated from the thermal stability assay data for **a**. $C_{12}E_9$, **b**. 10M and **c**. 9M. The cut-off threshold for reliable detergent exchange was set at 17 min as depicted by a dashed line.

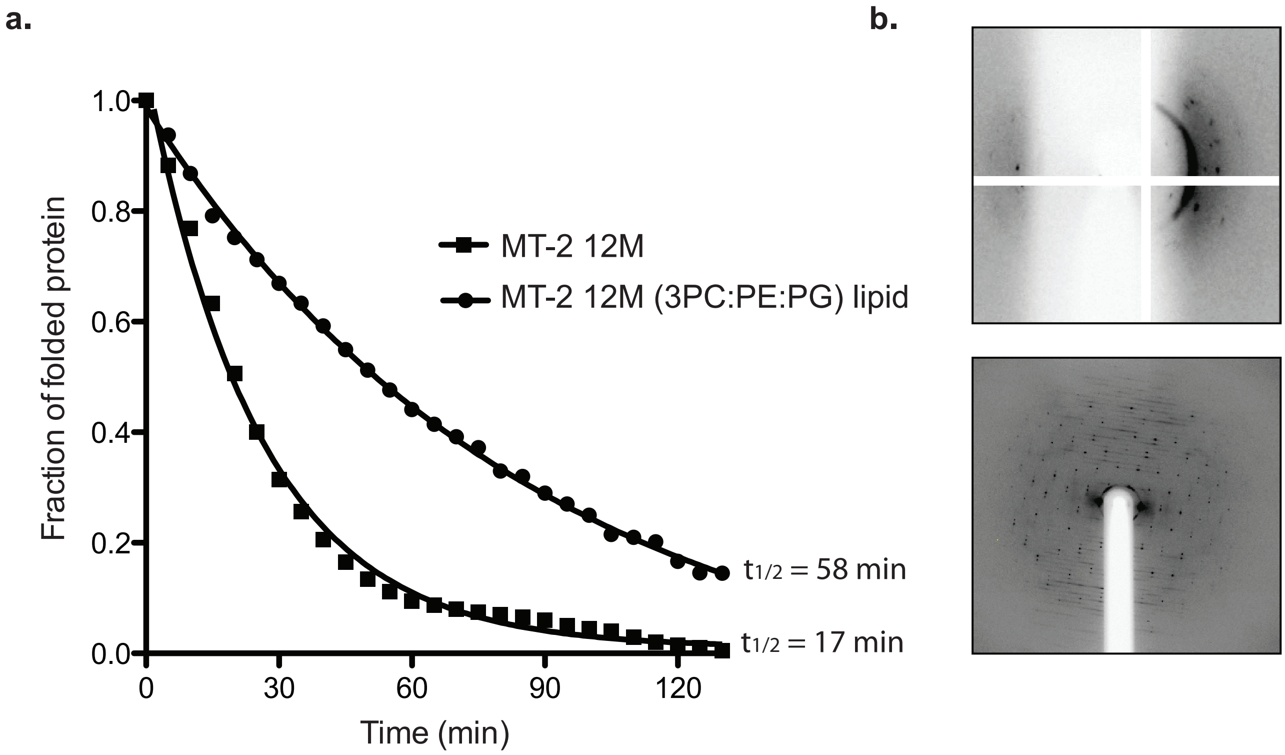

Figure SI 7a and 7b, related to Table 1,improving stability and crystal quality of MT-2

Figure 7, related to Figure 3. Stabilization and X-ray diffraction of the sugar transporter MT-2 is improved by addition of lipids

a. Unfolding rates for the sugar transporter, MT-2, from *R. norvegicus* in standard purification buffer (squares) and with the final addition of 0.1 mg/ml lipid phosphatidylcholine, phosphatidylethanolamine and phosphatidylglycerol in a ratio 3:1:1 to the purification buffer (circles). **b**. Improvement of X-ray diffraction from crystals of MT-2 using sample prepared in the absence (upper panel) and presence (lower panel) of additional lipids to the crystallization buffer.

a.

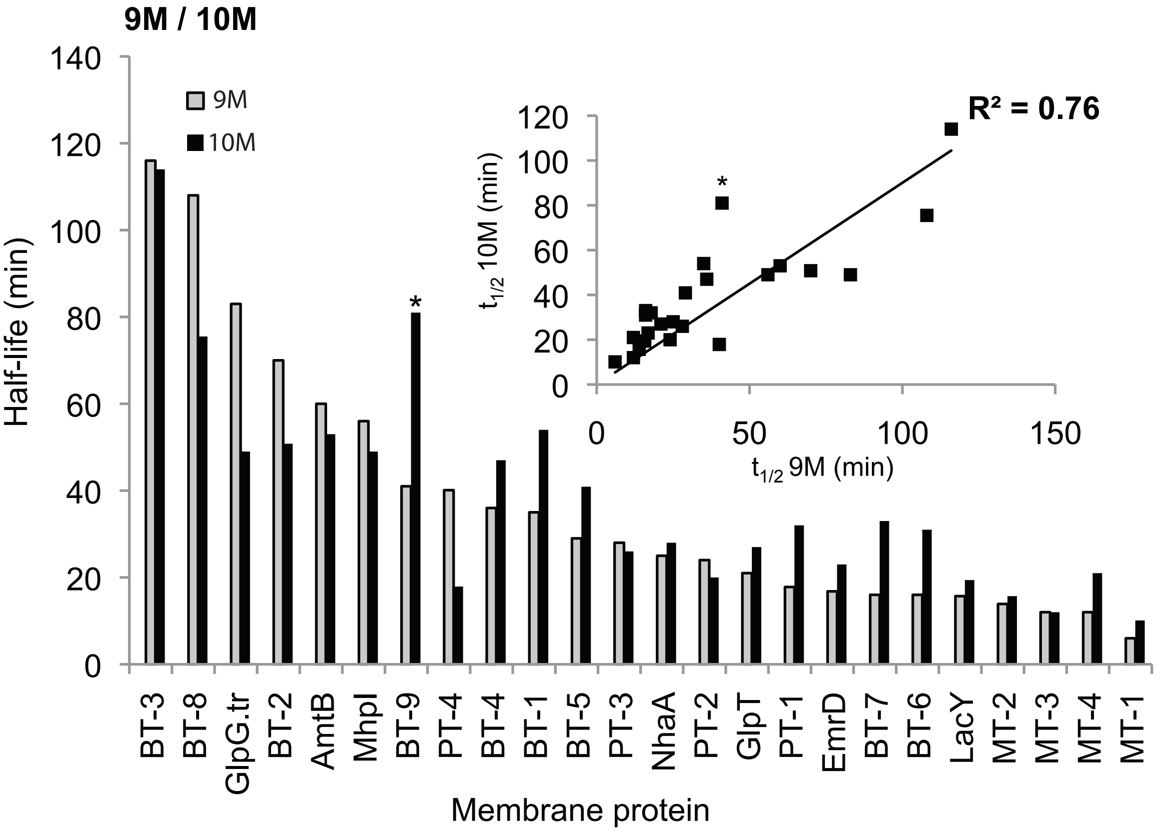

b.

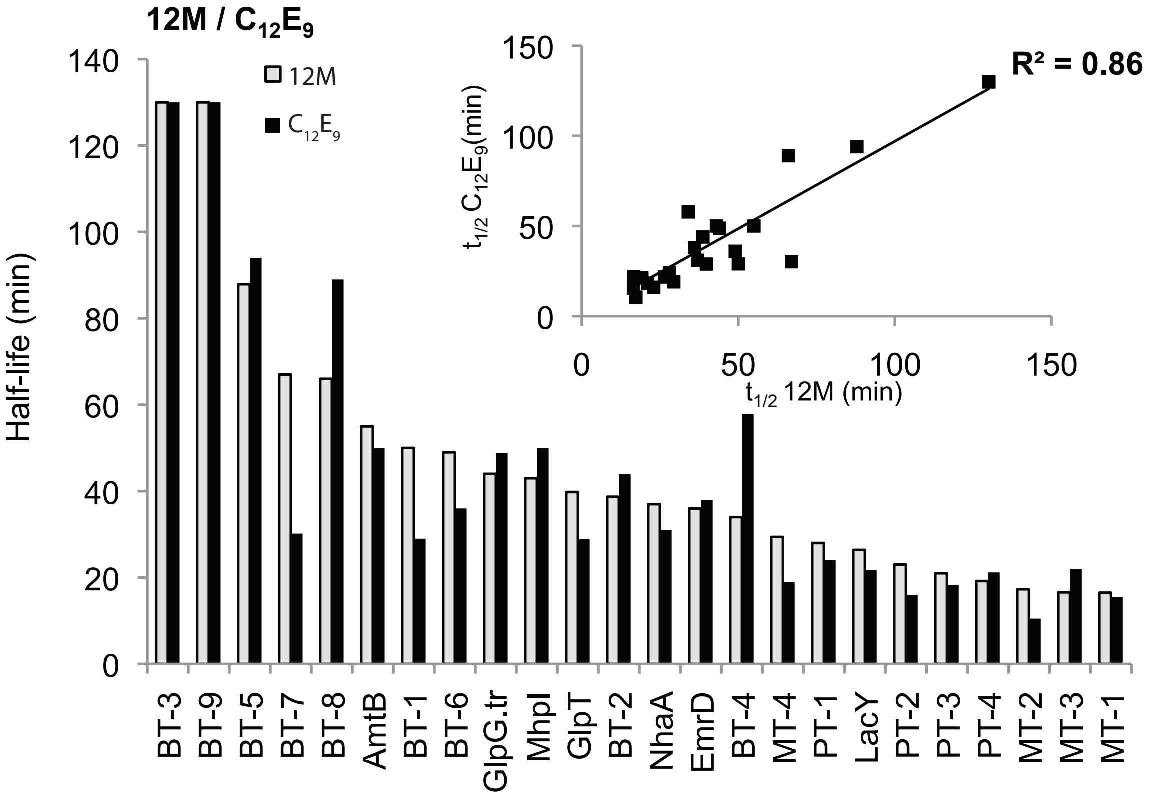

Figure SI 8a and b, related to Figure 3b and 3c, comparing the stability measured for each protein in different detergents

Figure 8, related to Figure 4. Membrane protein stability is predominantly intrinsic **a**. Bars represent the unfolding half-life for each protein in 9M (black bars) and plotted against that measured in 10M (grey bars); unfolding rates for 9M were plotted from the highest to lowest (left to right). Inset is a linear curve indicating the average stability difference between 10M and 9M. Asterisk for protein BT-9 indicates that we considered this difference as an outlier and, as such, was not included in calculation of the correlation co-efficient as displayed in inset. **b**. Bars represent the unfolding half-life for each protein in $C_{12}E_9$ (filled) and plotted against that measured in 12M (non-filled); unfolding rates for 12M were plotted from the highest to lowest (left to right). Inset is a linear curve indicating the average stability difference between 12M and $C_{12}E_9$. The > sign above BT-3, BT-9 bars indicates that the half-life although consistent in both 12M and $C_{12}E_9$, was longer than the time measured to assess stability.

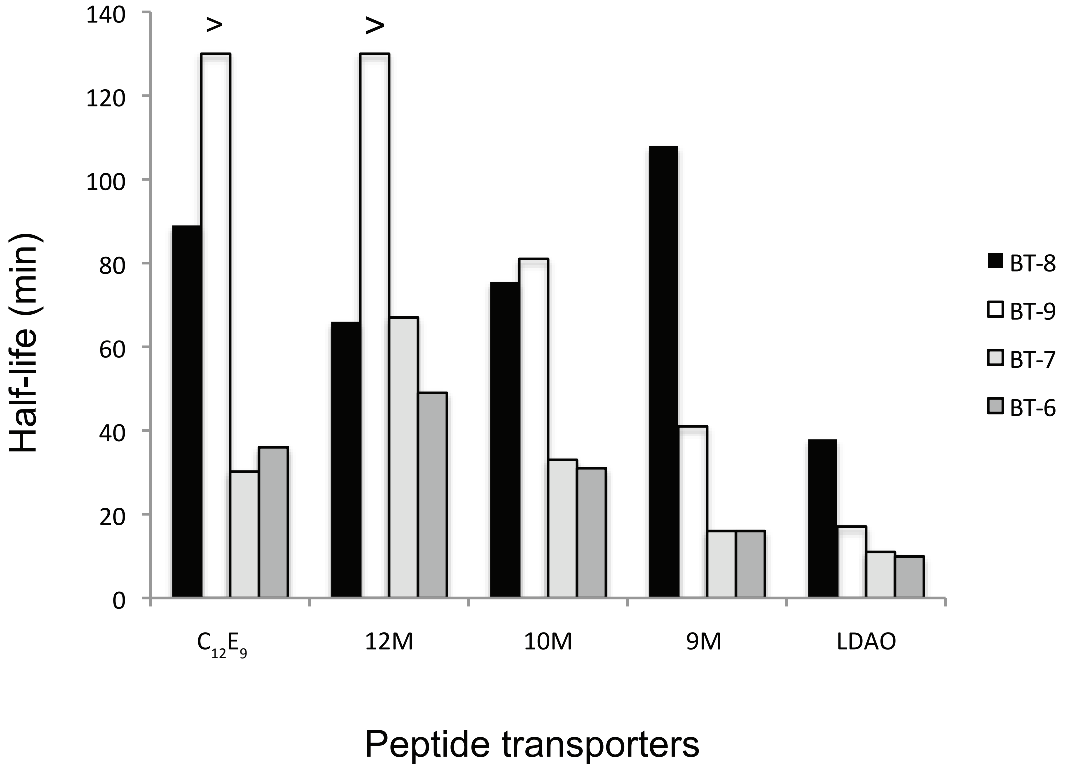

Figure SI 9, related to Table 1, selecting homologues for structural efforts based on stability

Figure 9, related to Table 1. Comparing stability of peptide transporter homologues
Bars represent the unfolding half-life for each peptide transporter in each of the detergents $C_{12}E_9$, 12M, 10M, 9M and LDAO. Black bars = BT-8, white bars = BT-9, light grey bars = BT-7 and dark-grey bars = BT-1. The > sign above BT-9 bars indicates that the half-life was longer than the time measured to assess stability.
